# Epidemiology of Valvular Heart Disease in Asia Pacific Region

**DOI:** 10.1016/j.jacasi.2025.03.011

**Published:** 2025-05-20

**Authors:** Kent Chak-yu So, Jonathan Yap, Guang-yuan Song, Karl Poon, Shih-Hsien Sung, Mann Chandavimol, Kentaro Hayashida, Duk-Woo Park, See-Hooi Ewe, Mi Chen, Vyanne Hei-tung Chan, Juri Iwata, Tarinee Tangcharoen, Paul Tern, Han-Su Park, Mirvat Alasnag, Yohei Ohno, Jimmy Kim Fatt Hon, Rohan Bhagwandeen, Minoru Tabata, Alex Pui-wai Lee, Hasan Jilaihawi, Dee Dee Wang, Gilbert H.L. Tang, D. Scott Lim, Thomas Modine, Yat-yin Lam

**Affiliations:** a Division of Cardiology, Department of Medicine and Therapeutics, Prince of Wales Hospital, Chinese University of Hong Kong, HKSAR, China; b Department of Cardiology, National Heart Centre Singapore, Singapore; c Duke-NUS Medical School, Singapore; d Interventional Center of Valvular Heart Disease, Beijing Anzhen Hospital, Capital Medical University, Beijing, China; e The Prince Charles Hospital, Brisbane, Australia; f Cardiovascular Research Center, College of Medicine, National Yang Ming Chiao Tung University, Taipei, Taiwan; g Division of Cardiology, Department of Medicine, Faculty of Medicine, Ramathibodi Hospital, Mahidol University, Bangkok, Thailand; h Department of Cardiology, Keio University School of Medicine, Tokyo, Japan; i Division of Cardiology, Asan Medical Center, University of Ulsan College of Medicine, Seoul, Korea; j Department of Radiology and Research Institute of Radiology, Asan Medical Center, University of Ulsan College of Medicine, Seoul, Korea; k Department of Structure Heart Center, Fuwai Yunnan Hospital, Chinese Academy of Medical Sciences, Kunming, Yunnan, China; l Cardiac Center, King Fahd Armed Forces Hospital, Jeddah, Saudi Arabia; m Department of Cardiology, Tokai University School of Medicine, Isehara, Japan; n National University Heart Centre, National University of Singapore, Singapore; o John Hunter Hospital, Newcastle, Australia; p Department of Cardiovascular Surgery, Juntendo University Hospital, Tokyo, Japan; q Smidt Heart Institute, Cedars-Sinai Medical Center, Los Angeles, California, USA; r Section for Structural and Valvular Heart Disease, NCH Heart Institute, Naples, Florida, USA; s Department of Cardiovascular Surgery, Mount Sinai Health System, New York, New York, USA; t Division of Cardiology, Department of Medicine, University of British Columbia, Vancouver, British Columbia, Canada; u Division of Cardiology, Department of Medicine, Division of Cardiology, University of Virginia, Charlottesville, Virginia, USA; v Bordeaux University Hospital, Bordeaux, France; w Central Medical, Hong Kong

**Keywords:** Asia Pacific, degenerative valve disease, diagnosis, epidemiology, rheumatic heart disease, valvular heart disease

## Abstract

Valvular heart disease poses a significant health burden in the Asia-Pacific region, with its epidemiology varying widely across countries caused by diverse socioeconomic and health care situations. Rheumatic heart disease remains prevalent, especially in low- to middle-income areas, while degenerative valvular diseases are emerging in developed regions caused by an aging population. Significant disparities in access to health care and intervention result in variable clinical outcomes. In the past decade, transcatheter interventions have revolutionized the management of patients with valvular heart disease globally. In the Asia-Pacific region, the uptake and development of transcatheter valvular interventions has been slow until recent years. Continued collaboration across the Asia-Pacific region is essential to mitigate the impact of the upcoming surge of valvular heart disease in this diverse and rapidly changing area.

## Highlights

• VHD poses a significant and rising health care burden in APAC.
• Advancements in transcatheter valve interventions improve VHD management, but access disparities persist in APAC.
• Tailored diagnostic and treatment strategies are essential for optimal VHD outcomes in APAC.
• Collaboration in research and clinical practice is vital for addressing VHD in APAC.

Valvular heart disease (VHD) represents a significant and growing health care burden in the Asia-Pacific region (APAC), characterized by diverse etiologies, clinical presentations, and outcomes.^1^, ^2^, ^3^, ^4^ The epidemiology of VHD is influenced by a complex interplay of factors, including socioeconomic status, health care infrastructure, and cultural practices, leading to marked variations in disease prevalence and management across the region. Rheumatic heart disease (RHD) continues to pose substantial morbidity and mortality, particularly in low- to middle-income areas where preventive care and early diagnosis are still limited.^5^^,^^6^ On the other hand, degenerative valvular diseases, often linked to an aging population, are increasingly recognized in more developed areas.^1^, ^2^, ^3^, ^4^^,^^7^ Significant advancements in transcatheter valve interventions over the past 2 decades have revolutionized the management paradigm for patients with VHD globally.^8^, ^9^, ^10^ However, disparities in access to health care and new devices in APAC exacerbate the burden of disease, resulting in variable patient outcomes (Central Illustration). This review aims to provide a comprehensive overview of the current epidemiological trends of VHD in the APAC compared with the Western world. By highlighting the unique challenges and opportunities, we hope to strengthen collaboration across the APAC to mitigate the impact of the upcoming surge of VHD in this diverse and rapidly changing region.

**Central Illustration:**
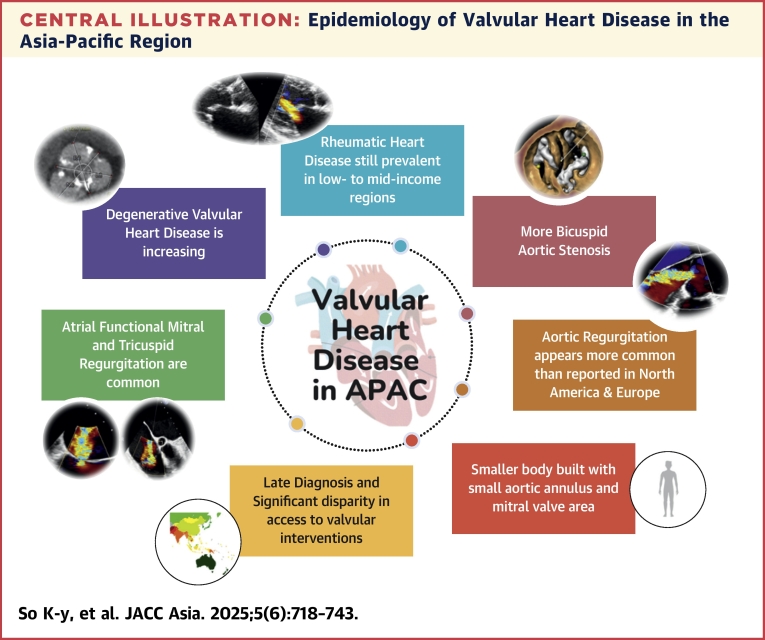
Epidemiology of Valvular Heart Disease in the Asia-Pacific Region In the Asia Pacific region (APAC), valvular heart disease has the following features: 1) rheumatic heart disease is still prevalence in low- to middle-income regions; 2) degenerative valvular heart disease is increasing because of the aging population, especially in high-income regions; 3) there are more bicuspid aortic stenosis than reported in North America and Europe; 4) there are more aortic regurgitation than reported in North America and Europe; 5) atrial functional mitral and tricuspid regurgitation are common; 6) Asians have a smaller body built with smaller aortic annulus and mitral valve area; and 7) there is a delay in diagnosis and significant disparity in access to valvular interventions in APAC, leading to different clinical outcomes.

## Rheumatic Heart Disease

### Pathology

RHD is caused by damage to heart valves following an abnormal immune reaction to group A streptococcal infections, typically contracted during childhood.^11^^,^^12^ The occurrence and prevalence of this condition exhibit considerable global variation, with the highest rates observed in low- and middle-income countries.^13^^,^^14^ The pathogenesis of RHD involves a complex immune response, both humoral and cellular, triggered by exposure to *Streptococcus pyogenes*, often following pharyngeal infection. Roughly one-half of all patients with acute rheumatic fever demonstrate inflammation within the heart, primarily targeting the valvular endocardium and potentially resulting in severe, irreversible valve damage,^15^ manifesting as irregular or localized thickening and restricted leaflet motion, eventually leading to heart failure or death. Early detection of asymptomatic RHD, particularly through echocardiographic screening of individuals with minimal valve abnormalities, is crucial for timely intervention.^16^^,^^17^

### Disease burden in APAC

**Figure 1:**
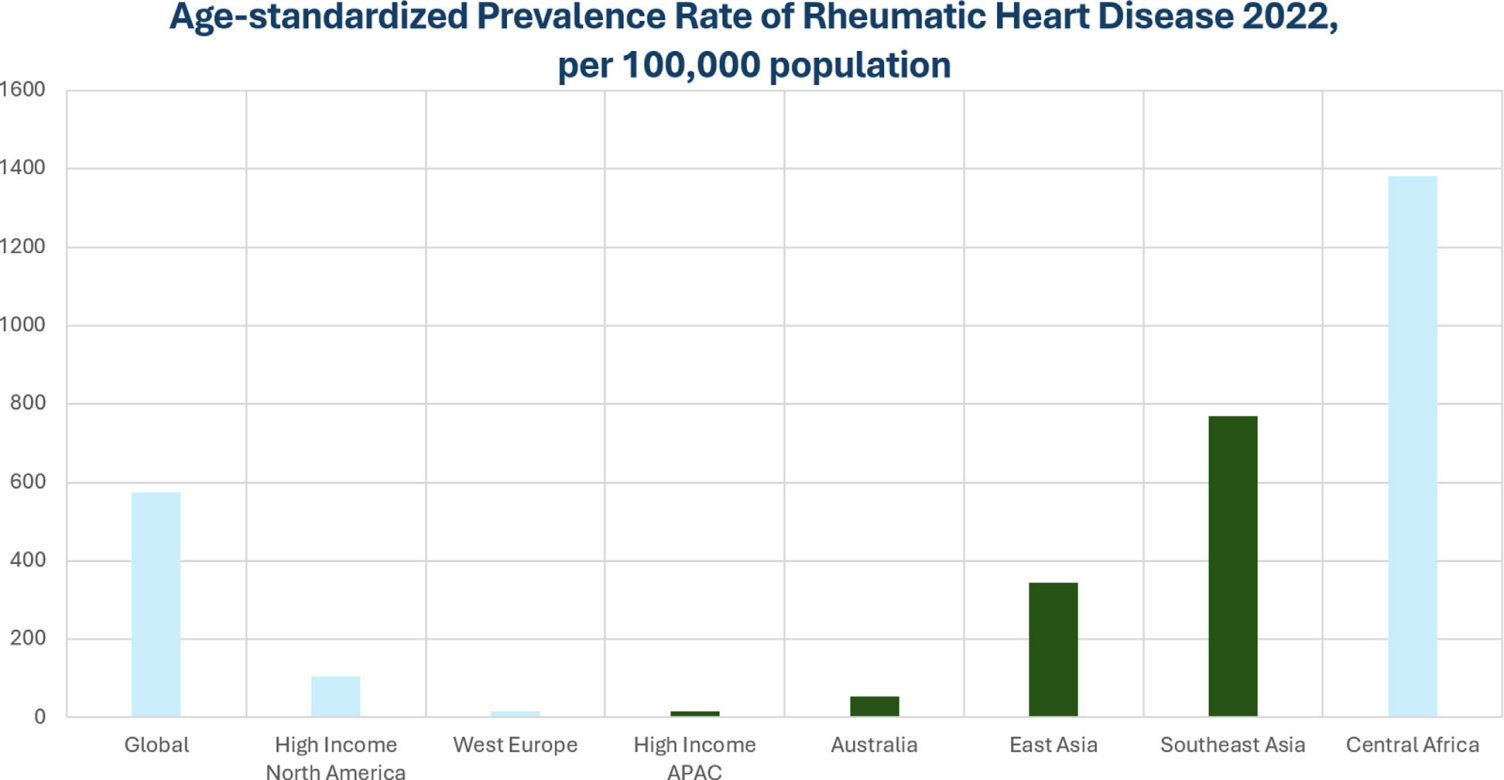
Age-Standardized Prevalence Rate of Rheumatic Heart Disease A significant difference in the prevalence of rheumatic heart disease was observed in the high income vs low- to middle-income Asia Pacific region (APAC) nations in the 2022 Global Burden of Cardiovascular Diseases Report.

Significant differences in the prevalence of RHD were observed across the APAC region in year 2022 Global Burden of Cardiovascular Diseases Report^18^ (Figure 1). Although the prevalence of RHD has declined in high-income countries,^5^^,^^18^ it remains a significant burden in low- to middle-income APAC nations. In the 2015 Global Burden of Disease Study, Watkins et al^5^ reported over 70% of the globe’s RHD were in India, China, and Pakistan. Chan et al^19^ additionally reported RHD as a significant social-economic disparity amongst under-represented communities such as the Maori and Pacific Islanders, and the Indigenous Australians (prevalence up to 15 per 1,000 in the Top End of the Northern Territory).

Rheumatic mitral valve disease remains the most prevalent and widely studied form of RHD worldwide. Other rheumatic valve conditions, such as rheumatic aortic stenosis (AS), are also common in APAC. The reported prevalence of rheumatic AS is 4.54 per 1,000 people in India, 1.86 per 1,000 in China, and 1.3 per 1,000 in Bangladesh. In higher-income APAC countries, such as Singapore, Japan, and South Korea, the prevalence is notably lower, ranging from 0.14 to 0.5 per 1,000 people.^20^ These numbers remain higher than the global average.

The RHD burden varies with age, showing higher prevalence among younger populations, peak incidence in young adults, and increased mortality among older adults. According to a retrospective study conducted in China, RHD remained the commonest cause of severe VHD in patients below 65 years of age.^21^ Despite the high disease burden, China experienced notable declines in the incidence, prevalence, mortality, and disability-adjusted life years associated with RHD between 1990 and 2019.^22^ Shi et al^23^ reported that the age-standardized incidence rate in China declined from 29.62 to 23.95 per 100,000, the age-standardized prevalence rate decreased from 446.15 to 390.24 per 100,000, and age-standardized mortality rate declined from 18.11 to 4.04 per 100,000, whereas the global rate decreased from 8.94 to 3.85 per 100,000. Despite this, Hu et al^24^ projected that by 2030, RHD prevalence still will exceed 48 million cases worldwide, with estimates indicating it will reach 35.75 per 100,000 in high-income regions of APAC and 300.84 per 100,000 in Southeast Asia, resulting in a continued economic burden.

Limited preventive measures and suboptimal adherence to penicillin prophylaxis have likely contributed to a continued rise in RHD prevalence, underscoring the need for targeted prevention efforts.^25^^,^^26^ Effective strategies to prevent or slow down RHD progression include community-level efforts to prevent Streptococcus pyogenes infections, and secondary prophylaxis with penicillin. Benzathine penicillin G remains the preferred antibiotic for secondary prevention. In low-income regions where RHD is endemic, the estimated rate of required cardiac surgeries is approximately 300 per million people annually, which exceeds the surgical capabilities in those areas.

## Aortic Stenosis

### Disease burden

AS is the most common VHD in industrialized countries.^1^ According to data from the United States and European Union, the prevalence of severe AS is approximately 7% in individuals over 80 years of age, compared with <1% in those under 70 years of age.^27^, ^28^, ^29^ The reported prevalence of AS appears to be lower in the APAC region than Western countries.^30^, ^31^, ^32^ A cross-sectional national survey from China involving 34,994 individuals age ≥35 years reported an overall AS prevalence of 0.7%, with the highest rates seen in those age ≥65 years (1.5% among those age 65-74 years and 3.4% among those age ≥75 years). Although East Asian ethnic groups are among the most populous (>1.5 billion people), data from Asian populations with severe AS are limited and the true disease burden of AS in elderly Asian individuals may be higher.^33^ Moreover, with the high prevalence of RHD, aging population, high prevalence of bicuspid aortic valve (BAV), and improving disease detection, the burden of AS is expected to increase in APAC.^34^

### Etiology

**Figure 2:**
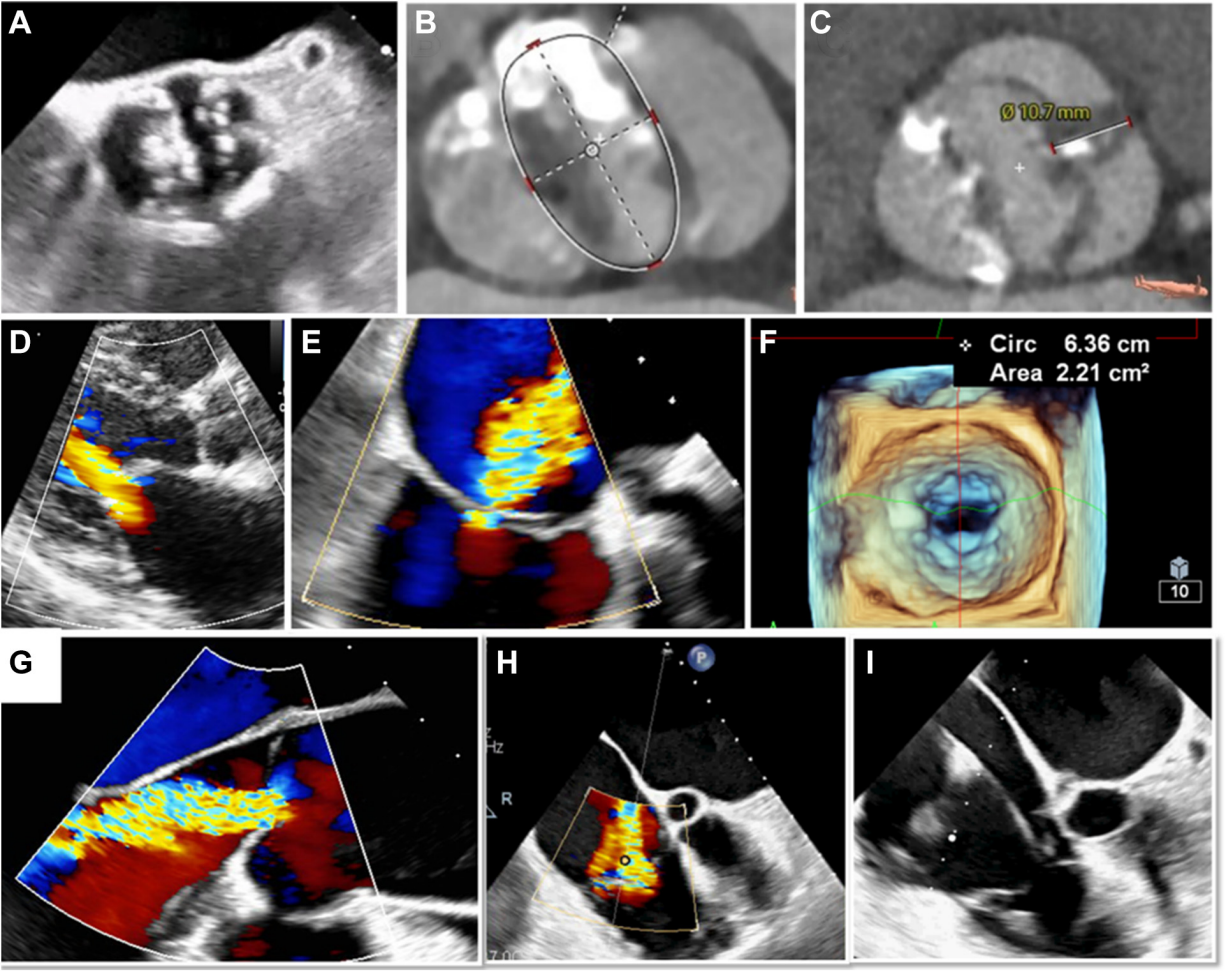
Features of Valvular Heart Diseases in the Asia-Pacific Region (A-C) Bicuspid aortic stenosis is commonly observed in the transcatheter aortic valve replacement population in the Asia-Pacific region than in Europe and the United States. In particular, type 1 (C) is found more frequently than type 0 morphology (B). (D) Rheumatic mitral stenosis remains prevalent in low- to middle-income regions. (E) Atrial secondary mitral regurgitation is increasingly recognized because of the high prevalence of chronic atrial fibrillation, which is characterized by late presentation and severe left atrial dilatation. (F) Patients with mitral regurgitation have smaller mitral valve areas caused by smaller body structures and mixed degenerative causes. (G) Aortic regurgitation is reported to be more common in Asia-Pacific region than in the United States and Europe. (H) Late presented atrial secondary tricuspid regurgitation with right atrial dilatation is common. (I) With the increasing use of cardiac implantable electronic devices, lead-related tricuspid regurgitation is increasingly recognized.

Although RHD remains prevalent in Low income regions in APAC, the main causes of AS vary depending on the region and era in which patients live. In more developed regions, where life expectancy is high, AS caused by degeneration associated with aging accounts for more than 80% of cases of severe AS. Rheumatic AS, which used to account for the majority of cases, is less commonly seen in developed countries in recent years because of the effective and timely treatment of rheumatic fever in pediatric patients. BAV is the most common congenital VHD with a prevalence of 0.5% to 2% in the general population and a male-to-female ratio of 3:1. It has also been reported that, among patients who undergo surgical aortic valve replacement before the age of 70, the number of patients with BAV is higher than the number of patients with tricuspid aortic valve.^35^ The prevalence of BAV varies slightly from study to study, depending on the definition used. It is generally defined as a spectrum of abnormal morphology in which the aortic valve has 2 functional cusps with fewer than 3 zones of parallel cusp apposition^36^ Chandra et al^37^ reported a racial difference in the prevalence of BAV, estimating frequencies of 0.17% in African-American patients and 1.1% in Caucasian patients *(P =* 0.001). Kong et al^38^ reported that Asians have a higher prevalence of type 1 BAV with a raphe between the right and non-coronary cusps than Europeans (19.7% vs 13.6%; *P* < 0.001), whereas Europeans have a higher prevalence of type 0 BAV (two commissures, no raphe) than Asians (14.5% vs 6.8%; *P* < 0.001) (Figure 2). Additionally, Asians with BAV have larger aortic dimensions than the Europeans counterparts. These findings have important implications for the selection of prosthesis type and size while considering transcatheter aortic valve replacement (TAVR).^38^^,^^39^

### Bicuspid Anatomy and Small Annulus

**Figure 3:**
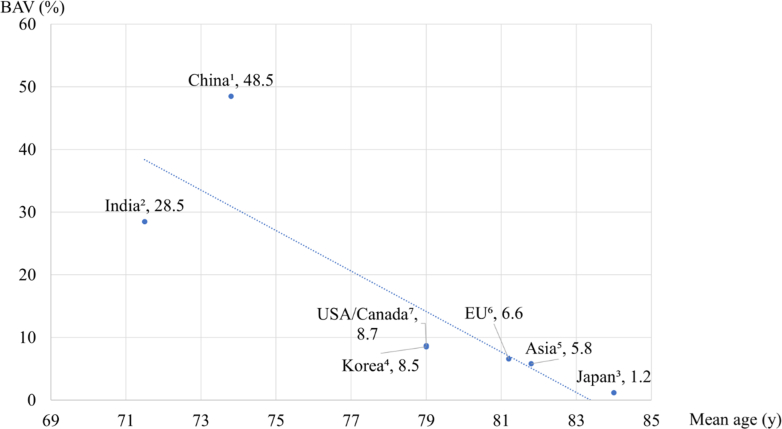
Features of Bicuspid Aortic Valve Undergone Transcatheter Aortic Valve Replacement The average age and proportion of bicuspid aortic valve (BAV) undergoing transcatheter aortic valve replacement in each region. (1) Li et al,^40^ (2) Datta et al,^41^ (3) Miyasaka et al,^44^ (4) Yu et al,^206^ (5) Yoon et al,^182^ (6) Maznyczka et al,^207^ and (7) Tang et al.^208^

Even within Asia, the proportion of patients with BAV who underwent TAVR varied significantly, higher in China (48.5%)^40^ and India (28.5%),^41^ where the average age for TAVR is lower. The age difference for patients receiving TAVR in different regions in APAC is affected by the variations in the length of healthy life expectancy, patients’ acceptance to open heart surgery, and level of medical care (Figure 3). The expanding indications for TAVR in younger patients suggest that the number of BAV patients will continue to rise. However, it is also reported that BAV patients may encounter a higher risk of cerebral ischemic events after TAVR.^42^ In APAC, it is crucial to clarify the safety of TAVR in patients with BAV and the prognosis of patients with small annuli, and to determine durability of small transcatheter aortic valves. Compared with Western patients, Asian patients have a lower body mass index, leading to smaller aortic valve area, higher mean pressure gradient, and smaller annular dimensions. Park et al^43^ demonstrated that in patients with severe AS who underwent TAVR, the incidence of prosthesis-patient mismatch was significantly lower in Asian patients than in non-Asian patients because of their smaller body surface area. The 1-year risk for the composite outcome of death, stroke, or rehospitalization was similar between the mismatch and no mismatch groups regardless of racial background. In addition, Miyasaka et al^44^ reported that the low prevalence of prosthesis-patient mismatch and 1-year mortality rate in patients with prosthesis-patient mismatch after TAVR implies that prosthesis-patient mismatch is not a significant risk factor for midterm mortality in Asian patients post-TAVR. Based on previous studies on prosthesis-patient mismatch and small annuli, it is predicted that the prognosis after TAVR does not differ significantly by annular size, especially in Asian cohorts, even when compared to Western populations. Over the past 2 decades, the indication for TAVR has been shifting towards younger patients.^45^ Therefore, a pre-operative computed tomography is advocated to devise the optimal management strategy, transcatheter versus surgery, taking into consideration the repeatability and lifetime management for Asian patients with BAV or small annuli.

## Aortic regurgitation

### Disease burden

Chronic aortic regurgitation (AR) is characterized by diastolic backflow from the aorta into the left ventricle (LV) caused by an incompetent aortic valve (Figure 2). It is arguably less recognized and more frequently undertreated^46^ compared to AS. Although AR can be associated with the severely restricted leaflets in AS—where there is no coaptation of leaflets—pure AR has a different pathophysiology and treatment implication to AS. On the other hand, acute AR from infective endocarditis, trauma, or aortic root dissection represents an emergency that can only be treated by surgical means, and should be recognized as a separate entity from chronic AR. The prevalence of moderate and severe chronic AR has been estimated from the Framingham study^47^ to be 4.9% and 0.5% respectively in a Western population aged 28 to 62 years. The prevalence was higher in male than female (13% vs 8.5%). Data specific to the APAC population are limited. In a study following Chinese elderly population undergoing aortic valve treatment,^30^ the prevalence of AR appeared higher than AS. Chinese patients with a BAV appear more likely to have moderate to severe AR compared to other ethnicity according to a Singaporean based study.^48^ Similarly, the China-DVD (China Elderly Valve Disease) study revealed that AR was more common than AS.^4^ Contemporary studies from Korea^3^ and Japan^2^ suggest that patients with AR requiring treatments tended to be younger than those with AS. This may reflect an observational bias related to the availability of transcatheter options for older patients with AS, whereas options for elderly patients with AR may be limited in the past.

### Etiology

Across various regions, the most common etiology for AR, in order of frequency, are degenerative causes, followed by functional or aortic dilatation, and RHD in China, Korea, and Japan. In Japan, RHD was the commonest cause of AR in the 1990s, but has since been surpassed by degenerative causes.^49^ The shift may be attributed to longer life expectancy and decreasing incidence of RHD in many regions. Yet, little data exist specifically for Pacific Islanders^6^ and limited-income countries, where RHD is comparatively more common. Moreover, there are unique anatomic differences between the Asian and Western populations, as well as among different Asian ethnicities. Although specific data for pure AR is limited, insights from studies on BAV reveal that aortic annulus and ascending aorta^39^ were surprisingly statistically larger in Asians compared with Europeans. The anatomic difference between Asian and Western patients may impact on future transcatheter treatment options for AR.

### Interventional management

In terms of indication for intervention, current American^9^ and European^8^ guidelines recommend intervention for severe symptomatic AR and severe asymptomatic AR with evidence of LV dysfunction (LV ejection fraction <50%). The Japanese national guidelines^10^ mirror these recommendations but suggest a lower cutoff for LV dilatation (LV end-systolic diameter 45 rather than 50 mm).^50^^,^^51^ A multicenter cohort study led by Yang et al^52^ focused on Asian patients with chronic AR suggests that indexed LV parameters with a lower cutoff than Western guidelines could be used in discriminating patients with excess mortality risk. It was also suggested that earlier intervention thresholds (ie, indexed LV end-systolic diameter >20 rather than 25 mL/m^2^), particularly relevant for Asian patients with smaller body habitus^53^ Surgery remains the gold standard for treatment of AR according to international guidelines.^9^ The need for concomitant ascending aortic surgery depends on aortic dimensions and underlying genetic predisposition to aortic dissection, with no specific variation noted for Asian and Western patients.

**Figure 4:**
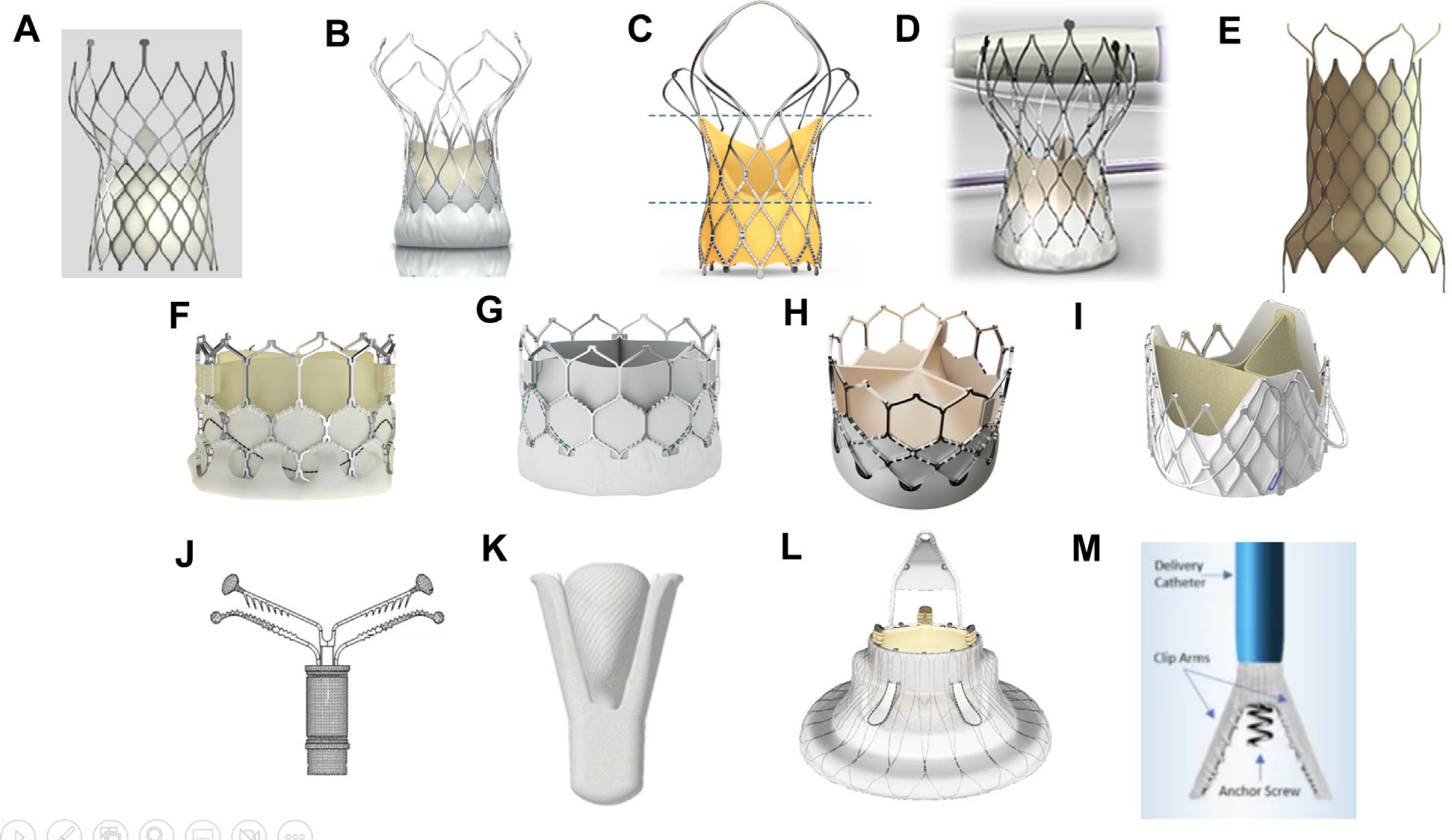
Selected Devices in Transcatheter Valvular Interventions From Asia Pacific Region (A) Self-expanding Venus-A transcatheter aortic valve replacement system (Venus Medtech).^a^ (B) Motorized retrievable self-expanding VitaFlow transcatheter aortic valve replacement system (MicroPort).^a^ (C) Self-expanding HYDRA transcatheter aortic valve replacement system (Vascular Innovations), with CE mark. (D) Self-expanding TaurusOne transcatheter aortic valve replacement system (Peijia).^a^ (E) Venus-P transcatheter pulmonary valve replacement system (Venus Medtech)^a^, with CE Mark. (F) Myval transcatheter aortic valve replacement system (Meril Life Sciences Pvt. Ltd), with CE Mark. (G) Balloon expandable Renatus transcatheter aortic valve replacement system (Balance Medical).^a^ (H) Balloon expandable Prizvalve transcatheter aortic valve replacement system (NewMed Medical Co).^a^ (I) J-valve transcatheter aortic valve replacement system for pure aortic regurgitation (JC Medical), apical system approved by China NMPA and transfemoral system under research. (J) ValveClamp Transapical Edge to edge repair system (Hanyu Medical Technology).^a^ (K) DragonFly Transcatheter Edge-to-Edge Repair system (Valgen MedTech).^a^ (L) LuX-valve Plus Transcatheter Tricuspid Valve Replacement system (Jenscare), under research. (M) K-Clip Focal Tricuspid Annuloplasty system (Huihe), under research. ^a^Approved by China NMPA.

Given significant undertreatment of AR, there is growing interest in less invasive transcatheter interventions for AR. Off-label use of conventional AS TAVR devices had been reported in Asian populations,^54^^,^^55^ yielding outcomes comparable to earlier American and European data^56^ in carefully selected patients. Nevertheless, recent studies indicate that off-label device use in native AR yields inferior results compared with dedicated AR devices,^57^^,^^58^ with significantly higher rates of embolization, need for second valve, and degree of residual AR. Two dedicated TAVR devices for AR were first implanted via a transapical approach.^59^^,^^60^ The transapical J-valve system (JC Medical Inc) was first approved in China in April 2017. Its transfemoral system was first implanted in human in 2018 in Canada,^61^ which features an 18- to 22-F transfemoral system that involves a 2-stage deployment process with the anchors are first deployed into respective sinuses, followed by the deployment of the self-expanding bovine pericardial THV (Figure 4). The compassionate registry in North America showed a reasonable procedure success rate (81%) and pacemaker rate (13%).^62^ The 30-day outcomes of the J valve Transfemoral China study (NCT05580952) showed a high technical success rate of 93.7% with low pacemaker rate of 9.5%^63^ in treating pure AR patients. The JenaValve device (JenaValve Technology), first implanted transapically in 2014,^64^ and also an 18-F transfemoral system (JenaValve Trilogy) involves a similar 2 stage deployment. The JenaValve Trilogy has been studied in the ALIGN-AR trial^65^ and is currently under U.S Food and Drug Administration review.

Regional variation of the prevalence of AR exists, and there may be a need for region-specific treatment threshold tailored to Asian patients. Surgical outcomes in developed Asian countries are comparable to high-volume centers in the United States. Dedicated transcatheter devices such as JenaValve Trilogy and J-valve, which was first implanted in China, hold promise for addressing the undertreatment of AR patients in Asia and other countries.

## Mitral Regurgitation

### Disease burden

Mitral regurgitation (MR) is the second most prevalent VHD, affecting approximately 3% to 6% of the global population.^1^ Its burden is particularly high among the elderly and is likely underestimated. The OxValve cohort study reported a prevalence of newly diagnosed significant MR at 2.3%, nearly 3 times higher than AS at 0.7%.^66^ Several factors contribute to the under-recognition of MR. First, its clinical symptoms are often less acute than those of AS. Patients with severe MR may present with subtle symptoms, such as exercise intolerance and lower leg edema, which may gradually progress over months or years to decompensated heart failure. Additionally, secondary MR, commonly associated with atrial fibrillation or cardiomyopathies with reduced systolic function, has historically not been regarded as a primary cause of cardiovascular mortality or morbidity. However, increased awareness and advancements in MR management have highlighted its significance, with MR accounting for 26.9% of cases in China^67^ and 35.6% in Japan.^2^

### Primary MR

Primary MR is caused by abnormalities in 1 or more of the valvular or subvalvular structures, leading to improper leaflet closure during systole. RHD remains a major cause of MR in South and Southeast Asia (Figures 1 and 2), although its prevalence is gradually declining caused by improvements in health care. Primary MR is also reported to be more common in young patients, whereas older patients are more prone to secondary MR.^21^ In developed countries, degenerative causes such as fibroelastic deficiency and Barlow’s disease are more common. Mitral annular calcification is also becoming prevalent among the elderly, impairing leaflet mobility and coaptation. A Japanese echocardiographic study reported that 14% of the population has mitral annular calcification, with 11.9% of these patients exhibiting significant MR.^68^

The importance of mitral repair and early intervention in improving outcomes for primary MR is well established.^69^^,^^70^ Although the rate of mitral valve repair is lower than that reported by the Society of Thoracic Surgeons Adult Cardiac Surgery database, the adoption of surgical repair and minimally invasive techniques is rapidly increasing in APAC.^71^^,^^72^ However, some studies have indicated that Asian patients with primary MR often remain untreated, leading to higher long-term mortality rates.^72^ This disparity may stem from prolonged intervals between diagnosis and treatment, influenced by cultural factors and the underestimation of volume overload when applying intervention thresholds derived primarily from Western populations. Additionally, Asian populations tend to have smaller anatomic features, a less thrombogenic coagulation profile, and increased renal vulnerability, which may further impact treatment approaches and outcomes^73^ (Figure 2). Although promising dedicated transcatheter mitral valve replacement devices are emerging, they are not yet widely available in the APAC region. Expanding treatment options for MR remains a critical goal.

### Secondary MR

**Figure 5:**
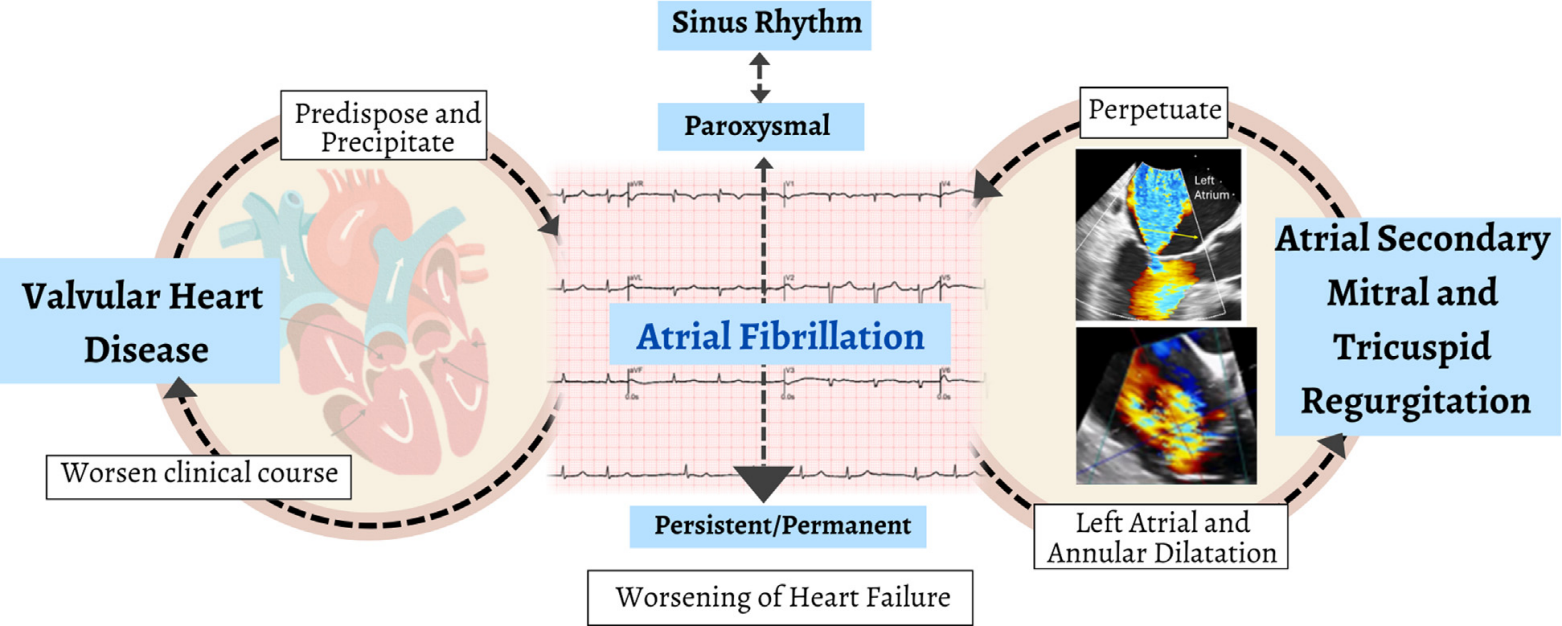
Interactions of Atrial Fibrillation and Valvular Heart Disease Progressive valvular heart disease predisposes individuals to and precipitates the development of atrial fibrillation, which results in worsening heart failure symptoms and an adverse clinical course. This progression increases the need for valvular interventions, while simultaneously promoting the development of persistent or permanent atrial fibrillation. Chronic atrial fibrillation leads to left and right atrial dilatation and mitral and tricuspid annulus dilatation, resulting in atrial secondary mitral regurgitation and tricuspid regurgitation. Nonetheless, mitral regurgitation and tricuspid regurgitation will perpetuate the development of persistent or permanent atrial fibrillation and exacerbate the severity of mitral regurgitation and tricuspid regurgitation.

Secondary MR arises from multifactorial dysfunction and remodeling of the left atrium or ventricle without structural abnormalities of the leaflets or chordae. This dysfunction leads to leaflet malcoaptation and subsequent MR. Determining the true prevalence of secondary MR is challenging because of variations across studies in patient selection, comorbid ischemic and non-ischemic etiologies, and differing methodologies.^74^ Recently, secondary MR has been classified into atrial secondary MR and ventricular secondary MR. Atrial secondary MR occurs in patients with preserved LV geometry, often caused by left atrial dilation, while ventricular secondary MR results from LV remodeling caused by cardiomyopathy (Figure 5). Although comprehensive prevalence data remain scarce, a retrospective echocardiography study in Australia found that atrial secondary MR accounted for 40% of secondary MR cases, whereas ventricular secondary MR comprised 60%.^75^ In APAC, we expect an increase in clinically significant secondary MR, particularly atrial secondary MR, caused by an aging population and rising prevalence of atrial fibrillation. Managing secondary MR requires a heart team approach, starting with optimal guideline-directed medical therapy. If significant MR persists despite medical management, transcatheter edge-to-edge repair procedures are recommended.^9^ Surgical intervention is typically reserved for patients requiring concomitant coronary artery bypass grafting or those ineligible for transcatheter-edge-to-edge repair. However, most patients with ventricular secondary MR are not ideal candidates for surgery. Although heart transplantation remains the definitive therapy for end-stage heart failure, its adoption is often limited in some regions caused by cultural and logistical factors. The OCEAN-Mitral (Optimized Catheter Valvular Intervention) registry, which recruited Asians, reported that secondary MR accounted for 75.2% of cases, with atrial secondary MR comprising 19.5% of the cohort.^76^ Similarly, the EXPAND G4 (Real-World Outcomes of Fourth-Generation Mitral Transcatheter Repair) global cohort that involved very small number of Asian included 57% secondary MR cases.^77^

### MR intervention in APAC

The first transcatheter mitral edge-to-edge repair procedure in Asia was performed in 2011. Data from the MARS (MitraClip Asia-Pacific Registry) showed early outcomes comparable to those in Western studies, with a procedural success rate of 93.7%.^78^ The OCEAN-Mitral registry reported an acute procedural success rate of 94.7%, demonstrating an excellent safety profile, with low incidences of complications. Notably, the clip sizes used in the OCEAN-Mitral registry differed significantly from those used in the global EXPAND G4 cohort caused by anatomic differences in valve size between Asian and Western populations, with more NT or NTW clips used in the former. In addition, smaller atria size in Asian population poses unique challenge for mitral edge-to-edge repair.^79^ Compared with a Western population, the reduced atrial dimensions hinder catheter navigation and device manipulation, complicating transseptal puncture and device implantation, often requiring more advanced steering for precise positioning and successful outcomes.^80^ Despite these anatomic differences, the procedural safety and clinical outcomes in Japan’s substudy analysis from the EXPAND G4 study yield comparable results.^77^^,^^81^ Novel devices originated from APAC, such as DragonFly-M device (Valgen MedTech), also demonstrated efficacy and procedural safety in treating patients with severe MR with prohibitive surgical risks.^82^^,^^83^

Despite the increasing recognition of MR interventions, treatment rates remain suboptimal, particularly among older patients because of surgical risks and comorbidities. Access to these interventions is often hindered by regional disparities in health care systems. For example, the out-of-pocket cost for transcatheter edge-to-edge repair therapy is approximately USD $1,500 in Japan compared to USD $37,000 in some other regions, exacerbating the burden of untreated MR in these regions. Overall, the landscape of MR in the APAC region highlights the necessity for increased awareness, improved access to innovative therapies, and tailored approaches to effectively manage and treat this prevalent condition.

## Mitral Stenosis

### Disease burden

Mitral stenosis (MS) imposes a significant disease burden, particularly in regions where RHD remains prevalent (Figure 1). Globally, RHD affects up to 30 million schoolchildren and young adults, with nearly one-third having MS, leading to substantial morbidity and disability.^84^^,^^85^ In India, a survey of rheumatic fever reported a mean presentation age of 15.1 years, with two-thirds of participants showing signs of MS, and one-half experiencing limiting symptoms.^86^ Although the prevalence of MS has decreased over time, the burden remains notable in certain regions. Similarly, in Korea, MS prevalence declined from 10.3% in 2007 to 3.6% in 2016.^87^ In developed countries, where RHD is largely controlled, the prevalence of MS detected by echocardiography ranges from 0.02% to 0.2%, underscoring the stark regional disparities in disease burden.^88^

### Etiology

Although MS most commonly results from RHD, fewer than one-half of affected patients remember having rheumatic fever. Persistent inflammation, valve damage,^89^ and hemodynamic stress contribute to the gradual progression of the disease. The rate of progression and time to clinical detection are strongly associated with repeated episodes of rheumatic fever, which may partly explain the varying natural history of MS across different regions of the world.^90^^,^^91^ Degenerative MS caused by mitral annulus calcification is an emerging concern, particularly in developed countries. The Euro Heart Survey reported a prevalence of 12.5% for degenerative MS.^85^ In 6% to 8% of patients with severe mitral annulus calcification, who are often elderly or dialysis-dependent, calcium infiltration into valve leaflets leads to MS.^92^ These patients frequently present with atypical physical findings, and surgical treatment options are often limited. In APAC, degenerative MS is less common. A report from Japanese region revealed a degenerative MS prevalence of 0.22%.^93^ Other rare causes of MS include congenital deformities, which typically present in infancy or early childhood (eg, parachute mitral valve, double orifice mitral valve, supra mitral ring^94^) and infiltrating diseases (eg, mucopolysaccharidosis); diseases affecting multiple systems (eg, Fabry’s disease, systemic lupus erythematosus, and rheumatoid arthritis) are very rare.

### Management

Closed mitral commissurotomy was one of the earliest procedures developed to alleviate RHD-related MS.^95^ However, open-heart surgery leads to a larger mitral valve area and improved cardiac function.^96^ Balloon mitral valvuloplasty has become the first-line treatment for suitable candidates with symptomatic MS because of its high success rate, low risk of restenosis, and minimally invasive nature. For patients ineligible for balloon valvuloplasty—such as those with high Wilkins scores, significant MR, or extensive commissural calcification, surgical intervention has been the option.^97^ In experienced Asian centers, rheumatic mitral valve repair has shown superior mortality rates and fewer valve-related complications compared with valve replacement.^98^ As an alternative, transcatheter options are under rapid evolvement for MS patients with prohibitive surgical risks, and transcatheter mitral valve replacement can be performed using either transcatheter aortic valves or dedicated devices.^99^ There are technical challenges that need to be overcome for successful transcatheter mitral valve replacement in APAC, eg, a small mitral annular size, hyperdynamic normal LV function, and small predicted LV outflow tract.

Atrial fibrillation is common among RHD patients with mitral valve disease, increasing stroke risk; however, anticoagulation therapy is often underutilized in low- and middle-income countries. Recently, transcatheter valve replacement has emerged as a transformative option for managing valvular heart disease, despite concerns about valve durability, especially in younger patients. Although research on biopolymeric valve leaflets suggest enhanced durability and reduced thrombogenicity,^100^ most current transcatheter valve designs are not optimized for RHD, who frequently present with subvalvular disease.

## Tricuspid Regurgitation

### Disease burden

The prevalence of tricuspid regurgitation (TR) in APAC is similar to the global trends. A national survey in China reported a TR prevalence of 0.8%, which is comparable to the 0.55% prevalence observed in the United States.^7^^,^^101^ Another retrospective analysis of 134,874 echocardiographic data revealed a higher prevalence for moderate to severe TR at 3.61%.^102^ A South Korea registry identified TR as the third most common VHD, affecting 28.6% of 4,089 patients.^3^ Similarly, a Japanese database found that 22.9% of 203,398 patients diagnosed with valvular heart disease had TR.^2^ Globally, the Framingham Heart Study identified age as an important determinant for TR, with prevalence of moderate to severe TR in individuals over 70-year-old 1.5% in men and 5.6% in women.^47^ Registries from United Kingdom and Europe also reported 2.6% to 2.7% prevalence of moderate or greater TR and right-sided VHD, respectively.^103^^,^^104^ There are variations in the reported prevalence in different studies, partially caused by differences in methodology, as well as the lack of standardization of echocardiogram quality and TR grading. Nonetheless, the disease burden of TR should not be overlooked, as the “forgotten valve” is gaining more recognition both in APAC and worldwide.

### Etiology

TR is broadly classified into primary and secondary TR. Primary TR involves structural changes to the tricuspid valve, including RHD and degeneration. Secondary TR is subcategorized into atrial secondary TR, related to chronic atrial fibrillation, ventricular secondary TR, caused by right ventricular dilatation, and cardiac implantable electronic device-related TR^105^ (Figure 2). In the APAC, etiology of TR mirrors global trends, with secondary TR being predominant. An echocardiographic study in China reported that 91% of TR cases were secondary, with the leading causes of severe functional TR being dilated cardiomyopathy (39.1%) and left sided VHD (21%).^102^ Similarly, Cleveland Clinic reported that 94.8% of TR cases were secondary, with the majority caused by left heart disease (54.4%) and atrial secondary TR (24.3%).^106^ There is growing recognition that atrial functional TR plays a significant role, with one-third of patients with atrial fibrillation developing moderate or greater TR (Figure 5), which independently worsens survival outcomes.^107^ Although primary TR is less prevalent in developed countries, RHD remains a significant contributor in low- to middle-income countries in APAC. The Global Burden of Disease Study 2015 reported the highest prevalence of RHD in APAC, including India, China, Pakistan (2.25 million), and Indonesia,^5^ with the highest mortality rates in South and East Asia. Although isolated rheumatic involvement of TV is rare, TR frequently accompanies rheumatic mitral and aortic valve disease.

### Prognosis

The survival rates of TR in the APAC are consistent with global data. China reported a 5-year survival rate of 77.01% for severe TR, comparable to the 70.6% survival rate observed in Kobe, Japan, and the 77.6% reported globally.^102^^,^^108^^,^^109^ Given the heterogeneity of TR patients, more recent studies have focused on identifying predictors of poor clinical outcomes and patients who benefit most from intervention. A study in Kobe, Japan, identified 4 prognostic factors for adverse outcomes, including death and heart failure hospitalization: 1) TR peak gradient >40 mm Hg; 2) blood urea nitrogen >25 mg/dL; 3) albumin <3.7 g/dL; and 4) left atrial volume index <34 mL/m^2^.^108^ Additionally, a U.S. study identified diabetes mellitus and a reduced LV ejection fraction as significant predictors of heart failure hospitalization.^110^ Other predictors of all-cause mortality include peripheral artery disease and an elevated INR (>1.2) without anticoagulation. Historically, medical management was the primary strategy for managing TR, but the global paradigm has shifted toward surgical and transcatheter interventions. However, isolated tricuspid surgeries are associated with risk and mortality.^111^ On this account, the TRI-score has been developed as a risk score model to predict in-hospital death in patients receiving isolated tricuspid surgery. First validated in a France,^112^ TRI-score was subsequently validated in the APAC population. In a Korean study, patients with higher TRI-score experienced a 50% increase in all-cause and in-hospital mortality and it also showed predictive power in long term prognosis in the Asian population.^113^

### Tricuspid innovations in APAC

In the APAC, the tricuspid transcatheter-edge-to-edge repair is still in its early stages, while transcatheter tricuspid valve replacement and focal annuloplasty techniques were developed from this region.^114^^,^^115^ Large-scale studies evaluating long-term outcomes of transcatheter intervention in this region are lacking. However, small-scale studies have begun comparing mortality and complication rates between transcatheter interventions and surgical approaches. A study in China involving of 262 TR patients who underwent surgical replacement, surgical repair, or transcatheter tricuspid valve replacement found significantly lower mortality rates in transcatheter tricuspid valve replacement patients (1.2%, vs 8.7% in surgical repair and 3.5% in surgical replacement). Transcatheter tricuspid valve replacement also showed lower rates of complications, including third-degree atrioventricular block, respiratory failure, and acute kidney injury.^116^ A separate retrospective analysis of 88 patients compared survival in patients with severe TR treated with transcatheter tricuspid valve replacement using the LuX-valve system versus medical therapy.^117^ The study found that patients undergoing transcatheter tricuspid valve replacement had a 2-year survival rate of 75.8%, compared with 48.4% in the medical therapy group, and experienced fewer heart failure hospitalizations. However, similar to the transcatheter mitral valve intervention, significant disparities exist in the access to different transcatheter tricuspid valve intervention in APAC because of different health care finance systems, expertise, and physician awareness. As transcatheter interventions continue to evolve in the APAC, future research should focus on identifying the optimal timing for these interventions, and the specific patient subgroups most likely to benefit from them.

## Congenital Valvular Disease

### Disease burden and distribution

Congenital heart disease (CHD) is the most common congenital anomaly, with reported incidences as high as 28%.^118^^,^^119^ The increasing availability of fetal echocardiography in tertiary care centers has made prenatal detection of CHD more accessible, allowing for early intervention and improved management strategies. Postnatal diagnosis using echocardiography has also become standard, enabling timely disease identification. These advancements in management have significantly increased survival rates over the decades, resulting in more patients with CHD surviving into adulthood and contributing to the growing population of adults with CHD. Global prevalence of CHD shows significant geographical differences. Recent large-scale meta-analyses reported that total CHD birth prevalence has increased substantially to 9.1 per 1,000 live births in the APAC region, largely caused by enhanced diagnostic capabilities.^120^^,^^121^ The prevalence of CHD is notably higher in Asia compared with other continents. Within Asia, various conditions, including pulmonary outflow obstruction and differing anatomical features, contribute to this trend. Differences may be partially attributed to genetic factors unique to populations in the region.^118^ A meta-analysis examining CHD in China between 1980 and 2019 revealed prevalence rates increased to 4.9 per 1,000 live births from 2015 to 2019, with variations influenced by socioeconomic contexts and monitoring methodologies.^122^

Differing from the Western population, pulmonary valve and right ventricular outflow tract anomalies represents a significant portion of CHD in Asia. According to a Korean study of CHD, pulmonary stenosis is the most prevalent, accounting for 24% of CHD cases, followed by Tetralogy of Fallot at 16%,^123^ highlighting their significance in this population. Targeting patients with pulmonary valve disease who require pulmonary valve replacement, transcatheter pulmonary valve replacement is an intervention of choice in the APAC region. The 2024 Statement from Asia expert operators on transcatheter pulmonary valve replacement has recommended transcatheter pulmonic valve replacement to be considered over surgical replacement when anatomically feasible,^124^ highlighting the region’s expertise and proficiency in performing the procedure.

Other than pulmonic valve disease, BAV also represents a significant subset of CHD. Research indicates that 7.9% of children under 18 years of age with CHD have BAV.^125^ In adults, echocardiographic studies have shown a prevalence of 0.43%.^126^ Patients with BAV often experience complications, including AS or AR. In a Korean study of mildly symptomatic BAV patients, 45% presented with moderate to severe AS or AR, showing disease progression over time.^127^ The need for regular monitoring is critical, particularly given that 74% of patients with moderate AS eventually progress to severe AS.^127^

### Challenges in CHD in APAC

Despite advances in interventional cardiology and treatments, challenges remain in managing CHD, especially in low- and middle-income countries within the APAC region. The ranking of CHD as a leading cause of death in children under one year has increased globally, rising from ninth to seventh between 1990 and 2017.^120^ In countries with a middle sociodemographic index, this condition has escalated from the fifth to the fourth leading cause of death during the same period.^120^

Challenges in managing CHD in APAC include financial and resource constraints, disparities between rural and urban health care systems, and a lack of awareness and education among health care providers. These factors can lead to delayed diagnoses and restricted access to treatment, ultimately resulting in suboptimal outcomes for patients.^128^^,^^129^ For example, patients with tetralogy of Fallot often experience treatment delays compared with their counterparts in Western countries, leading to adverse effects such as ventricular dysfunction and arrhythmias. In-hospital mortality rates following tetralogy of Fallot surgery are also higher in Asia, ranging from 1.9% to 10.2%, compared with 1.3% in the United States.

Improved patient longevity is creating further challenges, notably in managing the right ventricular outflow tract dysfunction, particularly in patients who have previously undergone surgical repair for conditions like tetralogy of Fallot. This often necessitates repeat interventions throughout patients’ lives. Innovations such as the increasing uptake of transcatheter pulmonary valve replacement show promise in addressing these challenges.^130^

To effectively manage congenital valvular heart disease in the APAC region, a multifaceted approach is essential. This includes increasing awareness and education, enhancing health care infrastructure, advocating for policy changes, and fostering global collaborations to improve outcomes for CHD patients in the long term. By ensuring that health care systems are equipped to handle the unique challenges presented by congenital valvular heart disease, we can better serve affected populations and improve their quality of life.

## Infective endocarditis

### Disease burden

Describing the trends in infective endocarditis (IE) in the APAC is challenging for several reasons. First, the region encompasses a heterogenous population, with countries at different stages of economic development. Moreover, rural-urban divides exist within countries, and accompanying socioeconomic variations have been shown to affect the presentation and outcomes of IE.^131^ Second, there is a lack of large-scale national or multinational IE registries, and most published literature are single-center reports based on small populations of patients and a certain specific clinical and geographical context. Also, large international surveys and registries such as the Global Burden of Disease Study and European Society of Cardiology (ESC) EURObservational Research Programme (EORP) EURO-ENDO (European Infective Endocarditis) registry^132^, ^133^, ^134^ lacks granularity on Asian population.

Gross incidence of IE has increased over the past 3 decades, with the Global Burden of Disease Study^132^ estimating a rise in age-standardized incidence rate from 10.22 per 10^5^ population in high-income APAC countries in 1990 to 12.84 per 10^5^ population in 2019. Correspondingly, age-standardized incidence rate also rose in Southeast Asia and Australasia from 9.60 per 10^5^ and 11.24 per 10^5^ population to 12.84 per 10^5^ and 16.46 per 10^5^ population, respectively, over the same period. IE has a male preponderance in APAC,^132^^,^^135^, ^136^, ^137^, ^138^ in keeping with similar observations in worldwide studies.^139^ The mean age of IE patients has risen over the years, with a corresponding increase in patient frailty and comorbidities.^135^^,^^138^ Significant comorbidities include diabetes, with incidence ranging from 13% to 29.7%,^140^^,^^141^ immunosuppression (4.3%-12.2%),^136^^,^^138^ and chronic hemodialysis (5.5%-10.5%),^137^^,^^138^^,^^142^ all of which have been shown to be associated with development of IE.^143^ When compared with contemporary Western studies, there is a relatively high burden of underlying RHD) (17%-42% in APAC,^140^^,^^144^^,^^145^ with greater incidence reported by hospitals in relatively less-developed regions. IE associated with intravenous drug use also varied greatly by region, with hospitals in cities such as Hong Kong^144^ and Kuala Lumpur^146^ reporting incidence of 22% to 30% amongst all patients presenting with IE, compared with 0% in a rural hospital in Northeastern Thailand.^145^

### Etiology

Overall, left-sided IE was still far more common than right-sided IE, with the mitral valve most affected (between 32.9% and 59%), followed by the aortic valve (27.6%-43.2%).^136^, ^137^, ^138^^,^^141^^,^^142^^,^^144^, ^145^, ^146^, ^147^ There was a wide range in incidence of prosthetic valve and cardiac implantable electronic device related IE, ranging from 6.7% to 15%,^135^^,^^138^^,^^141^^,^^145^^,^^147^ which may be confounded by small sample sizes but likely also reflects underlying differences in penetrance of these technologies. In the majority of studies, *Staphylococcus aureus* was the most common causative pathogen, accounting for up to 40.8% of all IE cases.^144^ However, a considerable number of studies also reported a high incidence of Streptococcus, and in particular of Streptococcus viridans,^137^^,^^141^^,^^144^^,^^145^ which is commonly found in oral flora. This was particularly so in studies originating from less-developed regions. In a study from a tertiary hospital in East China, Streptococcus accounted for 62% of all IE cases, with S. viridans the most common pathogen.^148^ This finding has implications for public health authorities, given the known association between poor oral hygiene and S. viridans IE, as well as the potential for preventative strategies with antibiotic prophylaxis under appropriate circumstances.^138^ Meanwhile, *Staphylococcus aureus* is known to be able to greatly increase its virulence by developing antimicrobial resistance, and indeed a worrying trend of increasing methicillin-resistant *Staphylococcus aureus* infections has been observed,^135^ accounting for up to 7.5% of all infections according to a large IE database in Japan.^141^ Several risk scores have been developed to predict risk of IE in patients with *Staphylococcus aureus* bacteremia. In a validation study from Singapore,^149^ the VIRSTA risk score^150^ performed well with patients having a score of <3 being able to be managed expectantly, with a negative predictive value of 97.5%.

### Management and outcomes

Despite advances in health care provision over the past decades, IE remains a condition with high morbidity and mortality. Reported in-hospital mortality ranges from 10.6% to 36.7%,^137^^,^^138^^,^^140^^,^^142^^,^^147^^,^^148^ with 1-year mortality in other studies reported to be between 11.3% and 32%.^135^^,^^138^^,^^147^ Nevertheless, there has been a small decrease in mortality across the years.^135^^,^^138^ Reported complications associated with IE included embolic stroke (10%-52.7%),^136^^,^^146^ other systemic embolism (30.7%-36.5%),^136^^,^^147^ acute heart failure (13.8%-52.8%),^138^^,^^147^ and valve perforation (14.7%-17.8%).^141^^,^^147^ Rates of surgical intervention in the region were generally high across the board (43.7%-75.8%).^136^, ^137^, ^138^^,^^141^^,^^145^^,^^147^^,^^148^ Studies from Malaysia (20%-32%)^140^^,^^146^ and Singapore (20.7%)^151^ reported slightly lower rates. Surgical intervention was associated with reduced mortality,^137^^,^^138^^,^^145^ although this finding may be confounded by selection bias where frailer patients were rejected as surgical candidates.

### Comparison with Western data

The availability of national/multinational registry data from the US and EU provides valuable information on overall incidence, clinical characteristics, and outcomes of patients with IE in these regions.^152^, ^153^, ^154^ However, as previously mentioned, there is a lack of such data in the APAC. The ESC-EORP EURO-ENDO registry^134^ includes data from predominantly Europe but also some non-European countries including some from the APAC, but these are usually not differentiated. From available data, the trends in demographics, presentation, management, and outcomes of patients with infective endocarditis in APAC were broadly similar when compared with the United States and EU (Table 1). However, a few key observations can be made.

1. Rates of IE associated with intravenous drug use have increased over the past few decades in the United States and EU,^152^^,^^153^^,^^155^ and these rates were similar to those reported by hospitals in cities in APAC, suggesting that this epidemic is common to many countries worldwide and more should be done by governments to address this.
2. Although underlying RHD remains an important risk factor in the APAC context, rates of RHD are very low in U.S. and EU cohorts.^133^^,^^156^ This is evidence of the extent to which RHD has been reduced in these places and will be a goal for low-income countries in the APAC to strive toward.
3. Similar to APAC countries, reports from EU countries were also mixed in terms of Staphylococcus or Streptococcus predominance,^153^ with developed countries reporting more *Staphylococcus aureus* infections,^157^ likely caused by a combination of better public health efforts toward oral hygiene and a greater proportion of health care–related IE. *Staphylococcus aureus* was uniformly predominant in the United States,^154^ with an alarming rise in methicillin-resistant *Staphylococcus aureus* over the years to ∼12%.^155^
4. When compared with large meta-analyses of IE in U.S. and EU populations,^153^^,^^154^ rates of surgical intervention in APAC appeared significantly higher. This could be an artefact of small sample sizes, but may also point to more advanced disease in the APAC cohort with larger vegetations, abscess, or valve disruption that cannot be managed by medical therapy alone.
5. Nevertheless, overall rates of IE related mortality were comparable between APAC, US and EU cohorts.

**Table 1:** Comparison Between Patients With Infective Endocarditis in Asia Pacific, United States, and Europe

	Asia Pacific	United States	Europe
Age standardized incidence rate			
Values per 10^5^ population^a^	High-income: 12.54 Southeast Asia: 12.84 East Asia: 14.94 Australasia: 16.46	North America: 14.31	Western Europe: 18.06 Eastern Europe: 15.46 Central Europe: 12.28
Trend	↑	↑	↑
Rheumatic heart disease^b^	17-42	Not reported (likely because of low prevalence)	8.9
Intravenous drug use^b^	22-30 in cities	15.1-39.4	2.6-20.7
Predominant organism	*Staphylococcus* or *Streptococcus*	*Staphylococcus*	*Staphylococcus* or *Streptococcus*
Surgical intervention^b^	20-75.8	6.4-16.0	10.2-37.0
1-y mortality^b^	11.3-32	36.2-37.1	21.4-32.0

a Data obtained from 2019 Global Burden of Disease Study.^2^

b Values are estimated and represented as % of total population of patients with infective endocarditis.

The burden of IE in Asia Pacific is increasing, and it remains a condition with high morbidity and mortality. In low-income regions, RHD remains an important risk factor, and Streptococcus predominates, possibly reflecting poor dental health in the population. Several of these risk factors are potentially addressable by public health interventions where efforts should be directed toward. The need for APAC specific multinational registries to provide pertinent regional data is important work for the future.

### Multivalvular Disease

Multivalvular disease—the coexistence of stenotic and/or regurgitant lesions, affecting 2 or more cardiac valves—has historically posed significant diagnostic and therapeutic conundrums. According to the Euro Heart Survey, multivalvular disease was present in 20.2% of patients with native valve disease and 14.6% of those undergoing valvular surgery.^158^ RHD was identified as the most frequent cause of multivalvular disease (51.4%), followed by degenerative valve disease (40.6%).^158^ Similarly, the CHINA-VHD study reported multivalvular disease in 47.4% of patients with RHD, highlighting its relative prevalence in limited-income countries and the APAC.^159^

Single-valve procedures that are less demanding and carry a lower risk than double- or triple-valve procedures should be considered for these individuals.^160^ Besides, decision-making for multivalvular patients is often complex and frequently necessitates referral to or consultation with a Comprehensive Valve Center.^161^ Different treatment pathways are possible with the emergence of transcatheter options, including concomitant multivalvular surgery, concomitant multivalvular transcatheter interventions, staged transcatheter interventions, or hybrid open and transcatheter interventions in a staged fashion. Patient-centered therapeutic decisions should be made by a multidisciplinary heart valve team, with integration of numerous factors to optimize clinical outcomes, including patient’s preference, centers’ expertise, and the local health care situation.

## Imaging in VHD and Interventions

Early access to cardiac imaging is key in the management of VHD, because prognosis depends on accurate assessment of the etiology and severity of VHD. Moreover, cardiac imaging and interpretation are critical for risk stratification, and aid in guide timely intervention for both surgical and catheter-based therapies. The unique health care landscape of APAC region is shaped by a variety of demographic, socioeconomic, and health care factors, making the access to advanced imaging modalities or expertise even more variable.

Echocardiography remains the primary imaging modality for diagnosing and assessing VHD in the APAC region because of its wide availability, cost-effectiveness, and high diagnostic accuracy. In this regard, transthoracic echocardiography is typically the first-line imaging technique for identifying valvular lesions, assessing the severity of valvular dysfunction and for serial surveillance. Its noninvasive nature and portability make it especially suitable for use in rural and resource-limited settings, where sophisticated health care infrastructure and expertise may be inadequate. A recent meta-analysis has shown that early echocardiographic population based screening identifies a significantly larger number of subclinical RHD cases (18.28 per 1,000) compared with screening using auscultation followed by echocardiography (2.79 per 1,000).^162^ This underscores the importance of echocardiographic screening in endemic populations, to identify at-risk individuals for secondary prevention. In fact, a small handheld echocardiography tool is able to improve detection of RHD when compared with cardiac auscultation alone and is a feasible, cost-effective screening strategy in resource-limited or remote regions.^163^ The application of artificial intelligence on electrocardiograms may be useful in this region for the mass detection of significant valvular heart disease.^164^

In urban centers with advanced health care systems, comprehensive echocardiographic examination plays a central role in providing detailed evaluation of the valve pathology, in terms of the underlying mechanisms and hemodynamic severity (Figure 2). In particular, 3-dimensional (3D) echocardiography offers superior accuracy compared with conventional 2-dimensional imaging, enabling precise appreciation of valve morphology and dysfunction, including a real-time or live image display of the valve lesions, critical for procedural planning, and assessment during and after interventions.^165^ On the other hand, dobutamine stress echocardiography is a valuable modality for assessing patients with AS and discordant grading in whom the actual severity of AS is uncertain (ie, indexed AVA is severe ≤0.6 cm^2^/m^2^ but low gradient/velocity [<40 mm Hg/<4 m/s]), particularly for patients with impaired LV function.^166^ Moreover, exercise stress echocardiography is also useful when patients’ symptoms are disproportionate to the resting hemodynamics because exercise testing may unmask the rise in gradients or regurgitations or pulmonary hypertension and provide an explanation for symptoms.^167^

As transcatheter structural heart interventions have gained popularity throughout APAC over recent years, the role of cardiac imaging cannot be overstated. Unlike open heart surgery where direct visualization is possible, success with catheter-based therapies is highly dependent on accurate imaging in patient selection and planning, as well as procedural guidance during transcatheter interventions. 3D transesophageal echocardiography is pivotal in procedural planning, because it permits detailed visualization of valvular lesion and its spatial relationship with neighboring structures that is critical for appropriate sizing and selection of devices. More importantly, during the procedure, it provides continuous real-time imaging of cardiac anatomy to guide and evaluate procedural outcomes and to detect any procedural complications.^168^ Recently, novel 3D techniques such as multiplanar reconstruction that are capable of unlimited plane combinations for thorough interrogation of the cardiac structures, and photorealistic imaging with transillumination will make the procedural guidance simpler and faster. After intervention, echocardiography remains the primary imaging modality for serial follow-up because of its noninvasive nature and is widely available.

**Figure 6:**
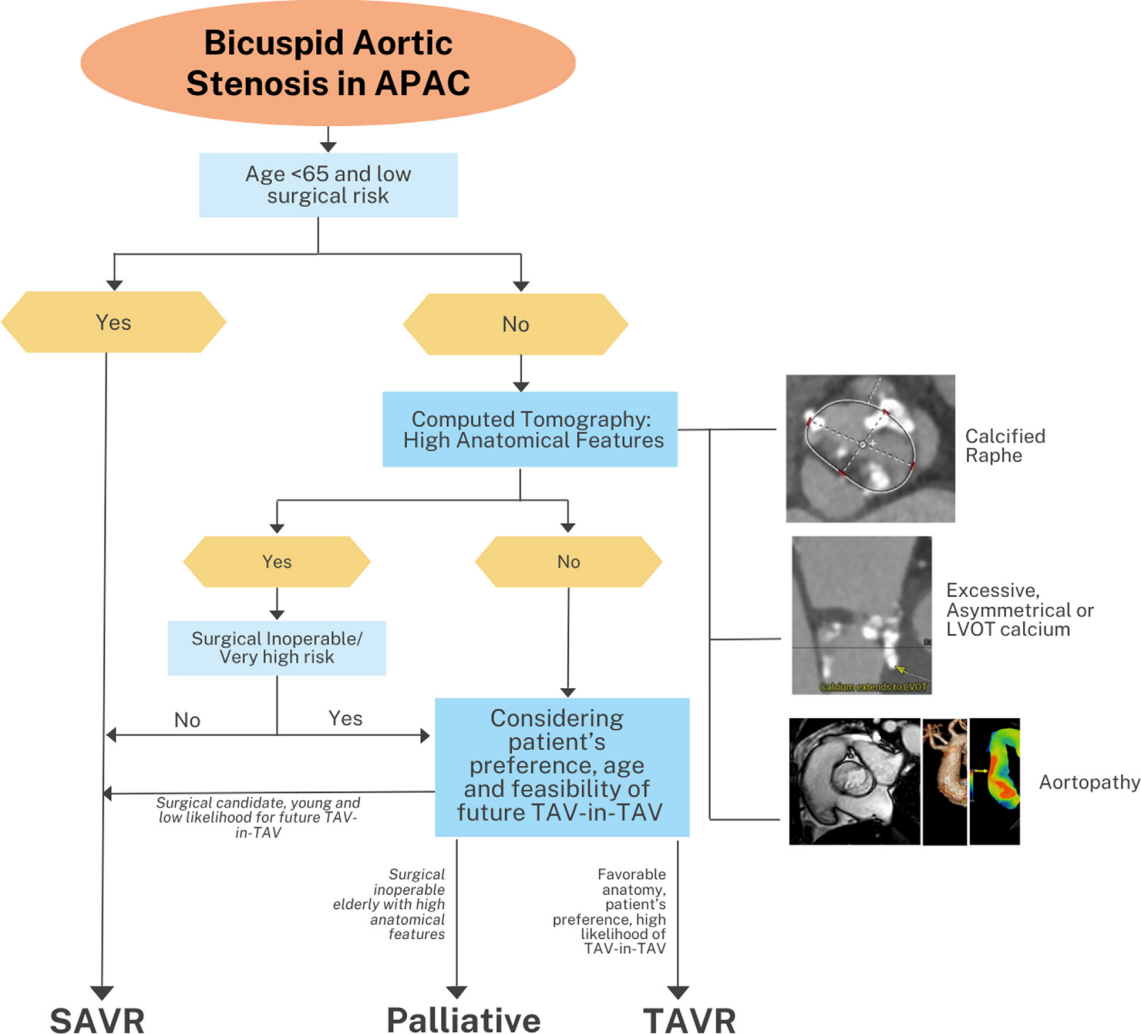
Proposed Algorithm to Manage Bicuspid Aortic Stenosis in APAC Bicuspid aortic stenosis is common in the transcatheter aortic valve replacement (TAVR) population. Surgical aortic valve replacement (SAVR) is preferred for patients aged <65 years with low surgical risk. For other patients, a preoperative computed tomography (CT) scan is recommended to assess the anatomy of the aortic root complex. If high-risk anatomical features (such as a calcified raphe, excessive, asymmetrical, or left ventricular outflow tract [LVOT] calcium, or concomitant aortopathy) are present, surgery is preferred unless the patient is at high surgical risk. The final decision should take into consideration the patient’s preferences, age, and the feasibility of future transcatheter aortic valve (TAV)-in-TAV procedures. If the patient is young and the likelihood of future TAV-in-TAV is low, surgery should be considered if the patient is a candidate. If the patient is surgically inoperable with high-risk anatomical features, palliative therapy may be considered. APAC = Asia Pacific region.

Besides echocardiography, computed tomography is essential in the selection of patients for suitability with various transcatheter aortic, mitral, or tricuspid valve devices. Although it was initially used for assessment of peripheral vascular access, computed tomography is now the gold standard modality for measurement of annular sizing and selection of transcatheter valve prostheses because of its high spatial resolution, allowing for detailed definition of the cardiac anatomy obtained from 3D volumetric data sets. In addition to identification of high-risk anatomy such as bicuspid aortic valve, severe calcification at landing zone, narrow aortic root or low coronary height, computed tomography provides optimal fluoroscopic projections in advance of the procedure that will reduce need for multiple root shots thereby reducing radiation and contrast usage and procedural time.^169^ It is important to highlight that the Asian population differs from Caucasians in key anatomical characteristics: smaller peripheral vessel sizes for vascular access, smaller aortic root sizes, and lower coronary ostia heights,^170^ as well as a higher prevalence of bicuspid aortic valve among the Chinese population, which is associated with greater calcium burden.^171^ All of these are concerning features when considering TAVR procedures, and therefore, a preprocedural computed tomography scan is the recommended modality to assess for feasibility of TAVR and procedural planning^166^ (Figure 6).

Although transesophageal echocardiography is the primary imaging modality used to support and guide transcatheter structural heart interventions, intracardiac echocardiography is becoming popular as an alternative to transesophageal echocardiography caused by several advantages. First, intracardiac echocardiography can be performed under conscious sedation without the need for endotracheal intubation and general anesthesia support, thus shortening procedural duration and fluoroscopy exposure and reducing hospital stays. Second, intracardiac echocardiography has superior image resolution, providing more detailed delineation of cardiac structures for near field-structures than those obtained by transesophageal echocardiogram. With the recent advent of 3D imaging capability (both biplane imaging and multiplanar reconstruction), 3D intracardiac echocardiography is indeed a major breakthrough for transcatheter tricuspid interventions because it provides equivalent or superior image quality to that of transesophageal echocardiography; tricuspid valve imaging on transesophageal echocardiography is often challenging because of various factors including a distant structure from esophagus and acoustic shadowing from intracardiac devices or cardiac structures (left sided heart valves).^166^^,^^172^^,^^173^ However, the widespread adoption of 3D intracardiac echocardiography in structural heart intervention remains a challenge in the APAC regions caused by the high cost of a single-use catheter and need for expertise and training in advanced imaging tools. Moreover, the current intracardiac echocardiography technology suffers from a limited field of view and inferior lateral resolution, limiting orthogonal plane imaging. However, 2-dimensional intracardiac echocardiography has been adopted in various parts of APAC regions and presents a cost-effective alternative to transesophageal echocardiogram in transcatheter closure of septal defect and left atrial appendage procedures.^174^

In conclusion, imaging plays a pivotal role in managing patients with valvular heart diseases, encompassing diagnosis, assessment of pathology and anatomy, preoperative evaluation/planning, and guidance during structural heart interventions. As the field of interventional imaging continues to evolve, there is a pressing need for structured curricula and training programs to ensure that structural imagers have the necessary expertise and competency to perform this complex task of providing rapid, accurate, high-quality image acquisition and interpretation in real time during interventions for best optimal procedural outcomes in APAC regions.

## Valvular Intervention

### Mechanical valve vs biologic valve usage

In APAC, the use of mechanical vs bioprosthetic valves for aortic valve replacement reflects global trends but is shaped by regional factors such as age demographics, health care accessibility, and economic status. According to the 2021 ESC guidelines for VHD, mechanical valve was recommended for patients under 60 years of age, whereas the bioprosthetic valve is suggested for those over 65 years of age.^175^ Mechanical valves have been widely used in younger patients because of their durability, especially in settings where reoperation may not be feasible. However, they require lifelong anticoagulation therapy.

Bioprosthetic valves, on the other hand, are preferred for older adults and gradually increasingly being used in middle-aged patients.^176^ This trend is influenced by factors such as the desire to avoid lifelong anticoagulation, the availability of valve-in-valve TAVR as an option in cases of structural valve degeneration, and advancements in technologies like sutureless and rapid deployment prostheses.^177^^,^^178^ These developments have expanded the use of bioprosthetic valves, particularly in patients who may benefit from less invasive reintervention options in the future.

### TAVR uptake and regional disparity

TAVR has evolved from being a treatment primarily for high surgical-risk patients to an option increasingly applied to those a lower surgical risk. Initially introduced in Western countries, TAVR has been widely adopted for severe AS.^179^ In Asia, the procedure was first performed in Singapore in 2009, however, its adoption has been slower compared with the United States and EU, particularly relative to the region’s large population.^32^ A major barrier to widespread TAVR adoption in many APAC countries is the high out-of-pocket cost, driven by insufficient medical reimbursement systems. The limited number of specialized centers and inadequate government support have restricted TAVR’s growth, especially in low- to middle-income APAC countries.^180^ These challenges have contributed to disparities in the adoption and outcomes of TAVR and other advanced medical devices across the region.

In most APAC countries, the majority of TAVR patients are elderly, aligning with global trends. Data from the OCEAN-TAVI registry and Asian TAVI registry indicated that the mean age of TAVR patients in Asia is similar with those reported in Western registries.^181^^,^^182^ Western guidelines including the guidelines from the European Society of Cardiology and American College of Cardiology recommend TAVR for patients above age of 75 and 80 years, respectively.^8^^,^^9^ Guidelines from the APAC region are consistent with these recommendations, with the Asian Pacific Society of Cardiology consensus similarly endorsing TAVR as the preferred treatment for patients aged 75 and older. For younger patients, those under 65 years of age, surgical aortic valve replacement remains the recommended approach caused by its durability and long-term outcomes.^166^ The Japanese Circulation Society draws no clear cutoff value for the age of TAVR and SAVR, but acknowledges age as an important parameter for prioritization, and suggest to consider TAVR in patients above 80 years of age.^10^

Baseline clinical, anatomic, and procedural characteristics reveal several differences between Asian and Western population. Surgical risk scores, such as those from the Society of Thoracic Surgeons scores, were similar in Asian and Western registries. However, Asian patients exhibited distinct anatomic features, including lower heights of the left coronary ostium, sinus of Valsalva, and sinotubular junction, and smaller aortic valve annulus and sinotubular junction compared with Western patients.^33^^,^^182^, ^183^, ^184^ Despite these anatomic challenges, procedure-related outcomes were comparable between the Asian and Western population. Data from Asian-TAVI registry and meta-analysis reported high procedure success rate (>95%). The recent AP-TAVI registry showed favorable clinical outcomes, including 30-day mortality (2.5%), 1-year mortality (8.8%), stroke (1.2%), and major vascular complication (5.8%).^183^ Similarly, findings from the OCEAN registry and Asian-TAVI registry aligned closely with results reported in Western registries.^181^^,^^182^

### Trends in non-TAVR transcatheter valve interventions

The transcatheter-edge-to-edge repair system, specifically the MitraClip (Abbott Vascular) was first approved for treating MR in the EU in 2008.^185^, ^186^, ^187^ Since then, its use has been extended widely across the EU. In early 2011, the transcatheter-edge-to-edge repair gained approval for commercial use in Australia, Malaysia, and Singapore. However, the initial adoption has been limited because of the high cost and lack of national reimbursement.

MARS demonstrated acute procedural success rates comparable to those reported in European studies with success rated of 93.7% in MARS, 94% in the German TRAMI study, and 85% in the Swiss MitraSwiss study.^188^, ^189^, ^190^ Major adverse events observed in MARS were also similar with those reported in the EVEREST II (Endovascular Valve Edge-to-Edge Repair Study) trial, with rates of 12.7% vs 15%, respectively.^190^^,^^191^ In the prospective, multicenter, single-arm study to confirm the reproducibility of the safety and efficacy of the MitraClip technology in Japanese patients (AVJ-514) trial conducted for regulatory approval in Japan, acute procedural success was achieved in 86.7% of cases, with no major adverse events reported.^192^ Similarly, Lee et al^193^ documented early experiences with the MitraClip system, demonstrating excellent outcomes. Their study reported a 95% acute procedural success rate, no periprocedural major adverse events, and a low 30-day mortality rate of 5%. These findings underscore the safety and efficacy of MitraClip in the Asian context, although further large-scale studies are needed to validate these outcomes across broader patient populations.

## Device Innovation in APAC

In the APAC region, TAVR has seen significant growth as a leading option for treating severe AS. Edwards Sapien (Edwards Lifesciences) and Medtronic Evolut (Medtronic) systems have long been established. Newer devices such as the Navitor valve (Abbott) and the ACURATE NEO2 valve (Boston Scientific) have emerged and have been widely extended. These innovations offer better adaptability to aortic anatomy variations, which are often seen in APAC populations. In addition to Western devices, several local TAVR systems have gained regulatory approval in APAC countries. For example, China has approved locally manufactured valves such as the Venus-A (Venus Medtech Inc) valve and VitaFlow valve (MicroPort).^194^ Similarly, India's MyVal (Meril Life Sciences Pvt. Ltd, Vapi) and Hydra (Vascular Innovations Co Ltd) valve systems have been developed (Figure 4).^195^

For mitral valve repair, the PASCAL system (Edwards Lifesciences) has emerged as a next-generation transcatheter-edge-to-edge repair system, providing an alternative for patients who are unsuitable for surgical repair besides MitraClip. The PASCAL system offers unique features, including a central spacer that helps reduce mitral regurgitation and paddles designed for gentle leaflet capture, ensuring a precise and atraumatic repair.^196^^,^^197^ Its steerability and controlled closure mechanisms provide advantages in treating complex anatomies, making it particularly relevant in diverse patient populations, including those in the APAC region. Another transcatheter-edge-to-edge repair system developed from China, Dragonfly (Valgen MedTech), which has a compressible atrial-side central filler and a mechanically locked arm angle between 0° and 45°, has shown to have high device success rate and safety profile.^83^

Tricuspid innovations from APAC like transjugular transcatheter tricuspid valve replacement (LuX-valve Plus, Jenscare) and focal annuloplasty (K-Clip, Huihe) has shown promising early experience^114^^,^^115^ (Figure 4). Other tricuspid technologies like transcatheter-edge-to-edge repair with the TriClip system (Abbott) or DragonFly-TR system (Valgen MedTech) and transcatheter bicaval valve implantation (TricValve)^198^, ^199^, ^200^ are also becoming available in APAC. These innovations face challenges in the Asian population because of anatomic differences and varying causes of TR, which may affect the success of the procedure and device selection. Although early data shows promising results in APAC, including comparable success rates to Western populations, there is a lack of extensive studies, particularly registry data and randomized controlled trials, focused on Asian patients. To address these gaps, further studies, including region-specific trials and industry-sponsored randomized studies for this population, are essential to ensure effective and equitable adoption of these devices in Asia.

## Accessibility of Structural Heart Interventions in APAC

As economic disparities exist between different countries in APAC, accessibility to structural heart interventions differ across regions. Countries with higher socioeconomic status show higher volume load for transcatheter valvular interventions. As of 2018, there are around 137 TAVR centers in Japan, vs 120 in other parts of Asia,^201^ showing a slanted development of transcatheter interventions, favoring countries with higher socioeconomic status. Another key determinant is the cost of the device and government reimbursement for procedures. The market prices of the same device can vary significantly across APAC. Besides, government reimbursement programs exist in only some APAC countries such as China, Japan and Korea, thereby improving the affordability of the devices, and enhancing the growth and development of structural heart interventions in these regions.

## Future Development

The future of VHD in the APAC region is promising yet complex, necessitating a multifaceted approach to effectively address the growing burden of the condition.

### Technology to enhance early diagnosis

A pressing need exists for new technologies that facilitate the early diagnosis of VHD. Innovative tools such as advanced diagnostic techniques, artificial intelligence, and remote monitoring can significantly enhance the detection of VHD and improve patient outcomes by allowing timely interventions, particularly in resource-limited settings. By adopting these approaches, health care providers in APAC can ensure prompt diagnosis and management, ultimately reducing the adverse impact of VHD on patient health.

### Consolidating heart failure programs

In addition to technological advancements, developing specialized heart failure programs is vital. Heart failure specialists play an essential role in managing patients with advanced VHD, particularly given the high prevalence of heart failure within this population. Establishing these specialized programs will ensure that patients receive effective, timely management. Heart failure specialists can navigate the complex interplay of comorbidities associated with VHD,^202^ significantly improving patient outcomes through tailored care, including optimal guideline-directed medical therapy, device therapy, transcatheter valvular intervention, LV assist devices, and heart transplants—particularly amid growing demands for managing functional MR and TR.

### Involvement of electrophysiologists

The role of electrophysiologists is also crucial in the management of VHD, especially considering the rising burden of atrial fibrillation and cardiac implantable electronic devices in this context.^203^^,^^204^ Effective management of atrial fibrillation in patients with VHD is essential, because atrial fibrillation can exacerbate symptoms and increase morbidity. It is now recognized as a significant cause of secondary MR and TR.^75^^,^^107^^,^^205^ Electrophysiologists provide valuable diagnostic and therapeutic insights through interventions like catheter ablation, which can restore sinus rhythm and help with the burden of atrial fibrillation. With advancements in cardiac implantable devices, individualized management of arrhythmias or heart blocks, conducted by electrophysiologists following transcatheter or surgical interventions, is becoming more common. Tailored pacing strategies may also reduce the risk of developing atrial fibrillation and further complications related to MR and TR.

### Multidisciplinary heart team

**Figure 7:**
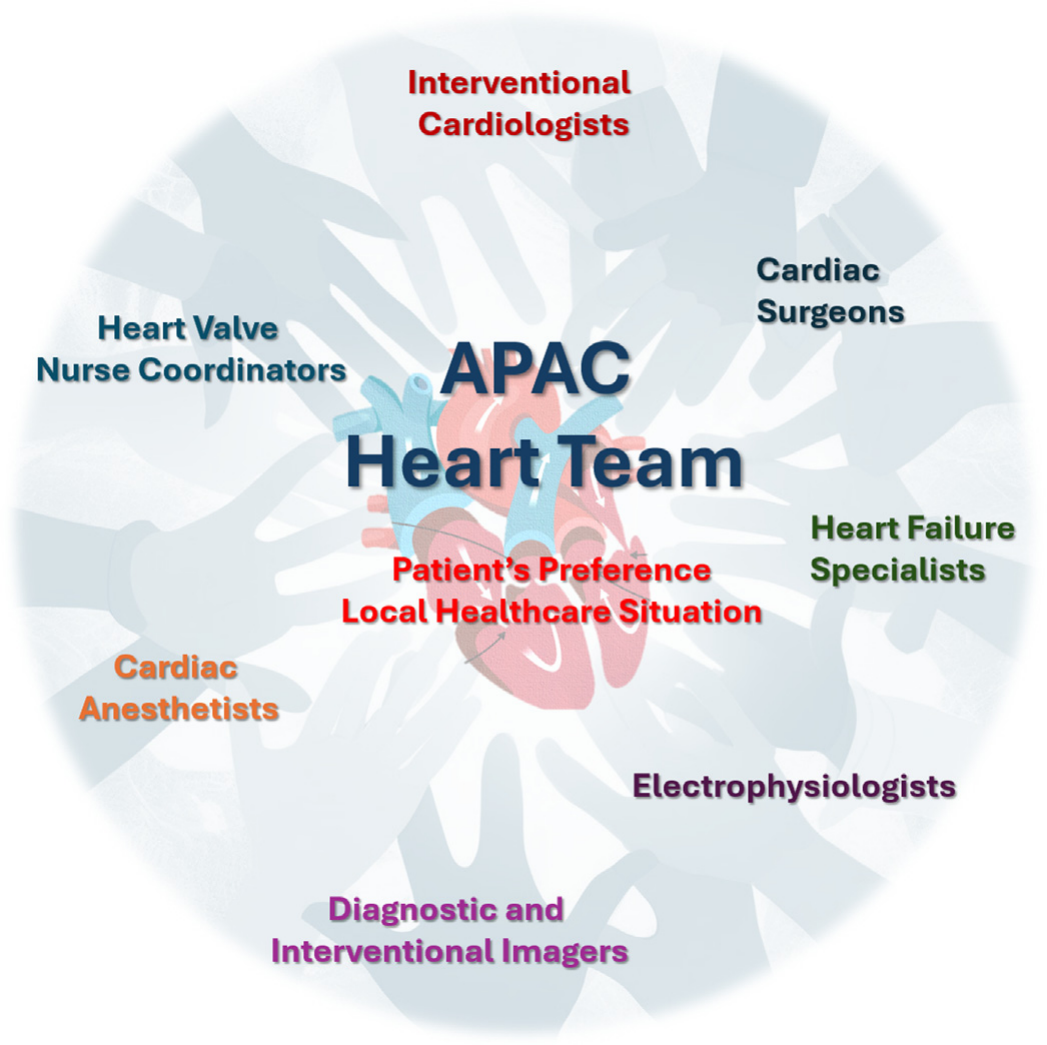
Multidisciplinary Heart Team in the APAC A multidisciplinary heart team approach to managing valvular heart diseases is advocated in the Asia Pacific region (APAC). The team should consist of interventional cardiologists, cardiac surgeons, diagnostic and interventional imagers, cardiac anesthetists, and heart valve nurse coordinators. Heart failure specialists and electrophysiologists should also be involved, especially in managing atrial fibrillation and cardiac implantable electronic device-related valvular heart disease.

Implementing a multidisciplinary heart team approach is vital for managing VHD effectively in the APAC region (Figure 7). This collaborative model integrates various specialists—such as interventional cardiologists, cardiac surgeons, heart failure experts, and electrophysiologists—to develop individualized treatment plans tailored to each patient’s unique clinical situation.^9^^,^^10^^,^^175^ This approach has been shown to improve patient outcomes, satisfaction, and treatment adherence by facilitating informed decision-making and offering a wide range of therapeutic options. The complexity of VHD in the region necessitates diverse health care professionals' expertise, because effective management often requires a holistic combination of medical management, structural interventions, and continuous follow-up care. Establishing formal heart team structures within hospitals and clinics across APAC will promote best practices and enhance care for patients with valvular conditions.

### Emerging role of artificial intelligence in valvular heart interventions

The development of artificial intelligence is expected to revolutionize VHD interventions globally and in the APAC region through procedural planning, and personalized treatment strategies. Computational modeling, such as DASI simulations and FEops HEARTguide for virtual procedure planning can facilitate preprocedure planning for structural heart interventions and enhance decision making especially in complex cases. With the huge burden and variety of VHD in APAC region, artificial intelligence potentially can play a pivotal role in improving health care quality and precision. However, challenges persist, particularly in lower-income APAC countries where access to advanced health technologies is limited. Additionally, regulatory frameworks are required for safe and effective integration of artificial intelligence into clinical practice in the long run.

### Clinical and research collaboration

The importance of clinical and research collaboration cannot be overstated in meeting the increasing demand for VHD management in the APAC region. The region's diverse populations and health care challenges present unique research opportunities that can be leveraged to inform clinical practices. Collaborative initiatives among hospitals, academic institutions, and industry stakeholders can foster an environment conducive to exploring new therapies, device innovations, and prevention strategies. Establishing regional registries and databases focused on VHD will facilitate the collection of critical data needed to drive research and improve patient care across the region. Partnerships among researchers, clinicians, industry professionals, and engineers can lead to effective translations of scientific discoveries into clinical applications, ultimately driving tailored solutions and dedicated guidelines on valvular diseases to meet the specific needs of APAC populations.

## Conclusions

VHD in the APAC presents a complex landscape characterized by significant challenges, including socioeconomic disparities and varying access to health care, alongside emerging opportunities for improved management through advancements in intervention techniques. The persistent burden of RHD highlights the critical need for comprehensive preventive strategies and timely interventions, particularly in low- to middle-income areas where health care resources are limited. As valvular diseases continue to evolve, the adoption of tailored diagnostic and treatment strategies, including region-specific solutions and innovative technologies, will be essential to address the unique anatomic and demographic characteristics of APAC populations, ensuring optimal outcomes for patients. Collaborative efforts across health care systems in APAC, coupled with increased research into regional epidemiology and treatment efficacy, are necessary to mitigate the rising burden of VHD and improve health outcomes in this diverse region.

## Funding Support and Author Disclosures

The authors received no financial support for the research, authorship, and/or publication of this paper. Dr So is a clinical proctor for Abbott, Boston Scientific, Edwards Lifescience, and Medtronic. Dr Yap has received speaker honoraria from Biosensors, Biotronik, Boston Scientific, Edwards Lifesciences, Johnson and Johnson, Kaneka, Medtronic, and Terumo. Dr Poon is a consultant for Edwards Lifesciences; has received institutional research grants from Abbott, Medtronic, and Edwards Lifesciences; and has received travel support from Edwards Lifesciences and Medtronic. Dr Chandavimol is a clinical proctor for Edwards Lifesciences, Boston Scientific, Abbott, and Medtronic. Dr Hayashida is a clinical proctor for Edwards Lifesciences, Medtronic, and Abbott. Dr Ewe has received speaker fees from Abbott Medical, Philips, and GE HealthCare. Dr Chen has received grants from Boston Scientific and Edwards Lifesciences; and is a consultant for Jenscare Scientific. Dr Ohno is a clinical proctor for Abbott and Medtronic. Dr Hon is a clinical proctor for Edwards and Medtronic; and has received speaker honorarium from Edwards and Medtronic. DrBhagwandeen is a proctor for Edwards Lifesciences and Medtronic. Dr Tabata is a clinical proctor for Medtronic, Abbott, and Edwards Lifescience; and has received a research grant from Medtronic and Abbott. Prof Lee has received a research grant from Abbott and Philips. Dr Jilaihawi has received an institutional clinical research grant from Pi-Cardia; is a consultant to Edwards Lifesciences and Medtronic; and is an investor in DASI simulations. Dr Wang is a consultant for Abbott, Boston Scientific, Edwards Lifesciences, Materialise, and NeoChord. Dr Tang has received speaker honoraria and served as a physician proctor, consultant, advisory board member, TAVR publications committee member, RESTORE study steering committee member, APOLLO trial screening committee member, and IMPACT MR steering committee member for Medtronic; has received speaker honoraria and served as a physician proctor, consultant, advisory board member and TRILUMINATE trial anatomic eligibility and publications committee member for Abbott Structural Heart; has served as an advisory board member for Boston Scientific; has served as a consultant and physician screening committee member for Shockwave Medical; has served as a consultant for Philips and Edwards Lifesciences, Peijia Medical and Shenqi Medical Technology; and has received speaker honoraria from Siemens Healthineers. Dr Lim has received consultant fees/honoraria from Ancora, Dinova Medtech, Valgen, Venus, W. L. Gore and Associates Inc; and is a coinvestigator in Abbott COAPT, REPAIR-MR and TRILUMINATE, Edwards CLASP IID/F, and Medtronic APOLLO clinical trials. Dr Modine has received administrative support, article publishing charges, statistical analysis, and writing assistance from Medtronic; has served as a consultant or advisor for Abbott, Medtronic, Microport, Edwards Lifesciences, and Jenscare Scientific Co; and has received funding grants from Medtronic and Edwards Lifesciences. All other authors have reported that they have no relationships relevant to the contents of this paper to disclose.

## References

[1] Santangelo G., Bursi F., Faggiano A. (2023). The global burden of valvular heart disease: from clinical epidemiology to management. J Clin Med.

[2] Izumi C., Matsuyama R., Asaoka M. (2023). Valvular heart disease in Japan: Characteristics and treatment of patients in acute care hospitals in 2019. J Cardiol.

[3] Choi Y.-J., Son J.-W., Kim E.K. (2023). Epidemiologic profile of patients with valvular heart disease in Korea: a nationwide hospital-based registry study. J Cardiovasc Imaging.

[4] Xu H., Liu Q., Cao K. (2022). Distribution, characteristics, and management of older patients with valvular heart disease in China: China-DVD study. JACC Asia.

[5] Watkins D.A., Johnson C.O., Colquhoun S.M. (2017). Global, regional, and national burden of rheumatic heart disease, 1990–2015. N Engl J Med.

[6] Mirabel M., Tafflet M., Noel B. (2016). Prevalence of rheumatic heart disease in the pacific: from subclinical to symptomatic heart valve disease. J Am Coll Cardiol.

[7] Yang Y., Wang Z., Chen Z. (2021). Current status and etiology of valvular heart disease in China: a population-based survey. BMC Cardiovasc Disord.

[8] Vahanian A., Beyersdorf F., Praz F. (2021). ESC/EACTS Scientific Document Group. 202 1 ESC/EACTS Guidelines for the management of valvular heart disease. Eur Heart J.

[9] Otto C.M., Nishimura R.A., Bonow R.O. (2021). 2020 ACC/AHA guideline for the management of patients with valvular heart disease: executive summary: a report of the American College of Cardiology/American Heart Association Joint Committee on Clinical Practice Guidelines. J Am Coll Cardiol.

[10] Izumi C., Eishi K., Ashihara K. (2020). JCS/JSCS/JATS/JSVS 2020 guidelines on the management of valvular heart disease. Circ J.

[11] Kaplan M.H., Bolande R., Rakita L., Blair J. (1964). Presence of bound immunoglobulins and complement in the myocardium in acute rheumatic fever. Association with cardiac failure. N Engl J Med.

[12] Carapetis J.R., McDonald M., Wilson N.J. (2005). Acute rheumatic fever. Lancet.

[13] Carapetis J.R., Steer A.C., Mulholland E.K., Weber M. (2005). The global burden of group A streptococcal diseases. Lancet Infect Dis.

[14] WHO Study Group on Rheumatic Fever and Rheumatic Heart Disease (2001 : Geneva, Switzerland) and World Health Organization (20 October - 1 November 2001). https://iris.who.int/handle/10665/42898.

[15] Kamblock J., Payot L., Iung B. (2003). Does rheumatic myocarditis really exists? Systematic study with echocardiography and cardiac troponin I blood levels. Eur Heart J.

[16] Carapetis J.R., Hardy M., Fakakovikaetau T. (2008). Evaluation of a screening protocol using auscultation and portable echocardiography to detect asymptomatic rheumatic heart disease in Tongan schoolchildren. Nat Clin Pract Cardiovasc Med.

[17] Marijon E., Ou P., Celermajer D.S. (2007). Prevalence of rheumatic heart disease detected by echocardiographic screening. N Engl J Med.

[18] Mensah G.A., Fuster V., Murray C.J.L., Roth G.A. (2023). Global Burden of Cardiovascular Diseases and Risks Collaborators. Global burden of cardiovascular diseases and risks, 1990-2022. J Am Coll Cardiol.

[19] Chan N.Y., Orchard J., Agbayani M.J. (2022). 2021 Asia Pacific Heart Rhythm Society (APHRS) practice guidance on atrial fibrillation screening. J Arrhythm.

[20] Aluru J.S., Barsouk A., Saginala K., Rawla P., Barsouk A. (2022). Valvular heart disease epidemiology. Med Sci.

[21] Hu P., Liu X.-B., Liang J. (2017). A hospital-based survey of patients with severe valvular heart disease in China. Int J Cardiol.

[22] Cui J., Guo X., Yuan X. (2022). Analysis of rheumatic heart disease mortality in the chinese population: a JoinPoint and age-period-cohort study. Int J Environ Res Public Health.

[23] Shi L., Bao C., Wen Y., Liu X., You G. (2023). Analysis and comparison of the trends in burden of rheumatic heart disease in China and worldwide from 1990 to 2019. BMC Cardiovasc Disord.

[24] Hu Y., Tong Z., Huang X. (2022). The projections of global and regional rheumatic heart disease burden from 2020 to 2030. Front Cardiovasc Med.

[25] Coffey S., Roberts-Thomson R., Brown A. (2021). Global epidemiology of valvular heart disease. Nat Rev Cardiol.

[26] Marijon E., Mocumbi A., Narayanan K., Jouven X., Celermajer D.S. (2021). Persisting burden and challenges of rheumatic heart disease. Eur Heart J.

[27] Danielsen R., Aspelund T., Harris T.B., Gudnason V. (2014). The prevalence of aortic stenosis in the elderly in Iceland and predictions for the coming decades: the AGES-Reykjavik study. Int J Cardiol.

[28] Osnabrugge R.L., Mylotte D., Head S.J. (2013). Aortic stenosis in the elderly: disease prevalence and number of candidates for transcatheter aortic valve replacement: a meta-analysis and modeling study. J Am Coll Cardiol.

[29] Martinsson A., Li X., Andersson C., Nilsson J., Smith J.G., Sundquist K. (2015). Temporal trends in the incidence and prognosis of aortic stenosis: a nationwide study of the Swedish population. Circulation.

[30] Pan W., Zhou D., Cheng L., Ge J. (2014). Aortic regurgitation is more prevalent than aortic stenosis in Chinese elderly population: Implications for transcatheter aortic valve replacement. Int J Cardiol.

[31] Jang S.Y., Park S.J., Kim E.K., Park S.W. (2022). Temporal trends in incidence, prevalence, and death of aortic stenosis in Korea: a nationwide population-based study. ESC Heart Fail.

[32] Tay E.L.W., Ngiam J.N., Kong W.K., Poh K.K. (2018). Management of severe aortic stenosis: the Singapore and Asian perspective. Singapore Med J.

[33] Lee C.H., Inohara T., Hayashida K., Park D.W. (2021). Transcatheter aortic valve replacement in Asia: present status and future perspectives. JACC Asia.

[34] Yi B., Zeng W., Lv L., Hua P. (2021). Changing epidemiology of calcific aortic valve disease: 30-year trends of incidence, prevalence, and deaths across 204 countries and territories. Aging (Albany NY).

[35] Roberts W.C., Ko J.M. (2005). Frequency by decades of unicuspid, bicuspid, and tricuspid aortic valves in adults having isolated aortic valve replacement for aortic stenosis, with or without associated aortic regurgitation. Circulation.

[36] Sievers H.-H., Schmidtke C. (2007). A classification system for the bicuspid aortic valve from 304 surgical specimens. J Thorac Cardiovasc Surg.

[37] Chandra S., Lang R.M., Nicolarsen J. (2012). Bicuspid aortic valve: inter-racial difference in frequency and aortic dimensions. JACC: Cardiovasc Imaging.

[38] Kong W.K.F., Regeer M.V., Poh K.K. (2018). Inter-ethnic differences in valve morphology, valvular dysfunction, and aortopathy between Asian and European patients with bicuspid aortic valve. Eur Heart J.

[39] Kong W.K., Regeer M.V., Poh K.K. (2018). Inter-ethnic differences in valve morphology, valvular dysfunction, and aortopathy between Asian and European patients with bicuspid aortic valve. Eur Heart J.

[40] Li Y.-M., Xiong T.-Y., Xu K. (2021). Characteristics and outcomes following transcatheter aortic valve replacement in China: a report from China aortic valve transcatheter replacement registry (CARRY). Chin Med J.

[41] Datta R., Bharadwaj P., Aggarwal N. (2022). Transcatheter aortic valve replacement in the developing world: lessons learnt and its implications for practice. Med J Armed Forces India.

[42] Fan J., Fang X., Liu C. (2020). Brain injury after transcatheter replacement of bicuspid versus tricuspid aortic valves. J Am Coll Cardiol.

[43] Park H., Ahn J.-M., Kang D.-Y. (2021). Racial differences in the incidence and impact of prosthesis-patient mismatch after transcatheter aortic valve replacement. JACC: Cardiovasc Interv.

[44] Miyasaka M., Tada N., Taguri M. (2018). Incidence, predictors, and clinical impact of prosthesis–patient mismatch following transcatheter aortic valve replacement in Asian patients: the OCEAN-TAVI registry. JACC: Cardiovasc Interv.

[45] Hibino M., Pandey A.K., Hibino H. (2023). Mortality trends of aortic stenosis in high-income countries from 2000 to 2020. Heart.

[46] Thourani V.H., Brennan J.M., Edelman J.J. (2021). Treatment patterns, disparities, and management strategies impact clinical outcomes in patients with symptomatic severe aortic regurgitation. Structural Heart.

[47] Singh J.P., Evans J.C., Levy D. (1999). Prevalence and clinical determinants of mitral, tricuspid, and aortic regurgitation (the Framingham Heart Study). Am J Cardiol.

[48] Chew N.W., Phua K., Ngiam J.N. (2022). Inter-ethnic differences in valvular dysfunction, aortopathy, and progression of disease of an Asian bicuspid aortic valve population. Heart Lung Circ.

[49] Matsumura T., Ohtaki E., Misu K. (2002). Etiology of aortic valve disease and recent changes in Japan: a study of 600 valve replacement cases. Int J Cardiol.

[50] Amano M., Izumi C., Imamura S. (2016). Pre-and postoperative predictors of long-term prognosis after aortic valve replacement for severe chronic aortic regurgitation. Circ J.

[51] Saisho H., Arinaga K., Kikusaki S. (2015). Long term results and predictors of left ventricular function recovery after aortic valve replacement for chronic aortic regurgitation. Ann Thorac Cardiovasc Surg.

[52] Yang L.-T., Lee C.-C., Su C.-H. (2023). Analysis of left ventricular indexes and mortality among Asian adults with hemodynamically significant chronic aortic regurgitation. JAMA Network Open.

[53] Yang L.-T., Lo H.-Y., Lee C.-C. (2022). Comparison between bicuspid and tricuspid aortic regurgitation: presentation, survival, and aorta complications. JACC Asia.

[54] Chiam P.T.-L., Ewe S.H., Chua Y.L., Lim Y.T. (2014). First transcatheter aortic valve implantation for severe pure aortic regurgitation in Asia. Singapore Med J.

[55] Soong E.L., Ong Y.J., Ho J.S. (2021). Transcatheter aortic valve replacement for aortic regurgitation in Asians: TAVR for aortic regurgitation in Asians. AsiaIntervention.

[56] Yoon S.-H., Schmidt T., Bleiziffer S. (2017). Transcatheter aortic valve replacement in pure native aortic valve regurgitation. J Am Coll Cardiol.

[57] Poletti E., Adam M., Wienemann H. (2024). Performance of purpose-built vs off-label transcatheter devices for aortic regurgitation: the PURPOSE study. JACC: Cardiovasc Interv.

[58] Poletti E., De Backer O., Scotti A. (2023). Transcatheter aortic valve replacement for pure native aortic valve regurgitation: the PANTHEON international project. JACC: Cardiovasc Interv.

[59] Zhu D., Chen Y., Zhang J., Hu J., Guo Y. (2015). Transapical implantation of a new second-generation transcatheter heart valve in patients with pure aortic regurgitation: a preliminary report. Interact Cardiovasc Thorac Surg.

[60] Schäfer U., Schirmer J., Niklas S., Harmel E., Deuschl F., Conradi L. (2017). First-in-human implantation of a novel transfemoral selfexpanding transcatheter heart valve to treat pure aortic regurgitation. EuroIntervention.

[61] Hensey M., Murdoch D.J., Sathananthan J. (2019). First-in-human experience of a new-generation transfemoral transcatheter aortic valve for the treatment of severe aortic regurgitation: the J-Valve transfemoral system. EuroIntervention.

[62] Garcia S., Ye J., Webb J. (2023). Transcatheter treatment of native aortic valve regurgitation: the North American experience with a novel device. JACC: Cardiovasc Interv.

[63] Lai W. (2024).

[64] Silaschi M., Conradi L., Wendler O. (2018). The JUPITER registry: one-year outcomes of transapical aortic valve implantation using a second generation transcatheter heart valve for aortic regurgitation. Catheter Cardiovasc Interv.

[65] Vahl T.P., Thourani V.H., Makkar R.R. (2024). Transcatheter aortic valve implantation in patients with high-risk symptomatic native aortic regurgitation (ALIGN-AR): a prospective, multicentre, single-arm study. Lancet.

[66] d'Arcy J.L., Coffey S., Loudon M.A. (2016). Large-scale community echocardiographic screening reveals a major burden of undiagnosed valvular heart disease in older people: the OxVALVE Population Cohort Study. Eur Heart J.

[67] Xu H., Liu Q., Cao K. (2022). Distribution, Characteristics, and Management of Older Patients With Valvular Heart Disease in China: China-DVD Study. JACC Asia.

[68] Okura H., Nakada Y., Nogi M. (2021). Prevalence of mitral annular calcification and its association with mitral valvular disease. Echocardiography.

[69] Lazam S., Vanoverschelde J.L., Tribouilloy C. (2017). Twenty-year outcome after mitral repair versus replacement for severe degenerative mitral regurgitation: analysis of a large, prospective, multicenter, international registry. Circulation.

[70] Suri R.M., Vanoverschelde J.L., Grigioni F. (2013). Association between early surgical intervention vs watchful waiting and outcomes for mitral regurgitation due to flail mitral valve leaflets. JAMA.

[71] Ma L., Li R., Jiang L. (2020). Contemporary trends in mitral valve surgery in East China: a 10-year experience. Asian Cardiovasc Thorac Ann.

[72] Lin H., Gong J., Wu Y., Zheng Z., Hou J. (2022). A comparative study on surgical treatment of valvular heart disease between high-volume cardiac centers in China and STS Data. J Cardiovasc Dev Dis.

[73] Hall Y.N., Hsu C.Y., Iribarren C., Darbinian J., McCulloch C.E., Go A.S. (2005). The conundrum of increased burden of end-stage renal disease in Asians. Kidney Int.

[74] Zoghbi W.A., Levine R.A., Flachskampf F. (2022). Atrial Functional Mitral Regurgitation: A JACC: Cardiovascular Imaging Expert Panel Viewpoint. JACC Cardiovasc Imaging.

[75] Moonen A., Ng M.K.C., Playford D., Strange G., Scalia G.M., Celermajer D.S. (2023). Atrial functional mitral regurgitation: prevalence, characteristics and outcomes from the National Echo Database of Australia. Open Heart.

[76] Saji M., Yamamoto M., Kubo S. (2023). Short-term outcomes following transcatheter edge-to-edge repair: insights from the OCEAN-Mitral Registry. JACC Asia.

[77] von Bardeleben R.S., Mahoney P., Morse M.A. (2023). 1-year outcomes with fourth-generation mitral valve transcatheter edge-to-edge repair from the EXPAND G4 Study. JACC Cardiovasc Interv.

[78] Yeo K.K., Yap J., Yamen E. (2014). Percutaneous mitral valve repair with the MitraClip: early results from the MitraClip Asia-Pacific Registry (MARS). EuroIntervention.

[79] Zemrak F., Ambale-Venkatesh B., Captur G. (2017). Left atrial structure in relationship to age, sex, ethnicity, and cardiovascular risk factors: MESA (Multi-Ethnic Study of Atherosclerosis). Circ Cardiovasc Imaging.

[80] Wong N., Yeo K.K. (2019). MitraClip in Asia—current adoption and regional data. Circ Rep.

[81] Morikawa T., Enta Y., Sakamoto T. (2024). 1-year outcomes of fourth-generation mitral transcatheter edge-to-edge repair in Japan from the EXPAND G4 study. JACC Asia.

[82] Liu X., Chen M., Han Y. (2022). First-in-human study of the novel transcatheter mitral valve repair system for mitral regurgitation. JACC Asia.

[83] Wang J., Liu X., Pu Z. (2024). Safety and efficacy of the DragonFly system for transcatheter valve repair of degenerative mitral regurgitation: one-year results of the DRAGONFLY-DMR trial. EuroIntervention.

[84] Waller B.F., Howard J., Fess S. (1994). Pathology of mitral valve stenosis and pure mitral regurgitation—part I. Clin Cardiol.

[85] Rheumatic fever and rheumatic heart disease (1988). Report of a WHO Study Group. World Health Organ Tech Rep Ser.

[86] Padmavati S. (2001). Rheumatic fever and rheumatic heart disease in India at the turn of the century. Indian Heart J.

[87] Lee H.J., Cho I., Kim D.Y. (2024). Shifts in Clinical characteristics, treatment, and outcome for rheumatic mitral stenosis: insights from a 20-year multicentre registry study in Korea. J Korean Med Sci.

[88] Nkomo V.T., Gardin J.M., Skelton T.N., Gottdiener J.S., Scott C.G., Enriquez-Sarano M. (2006). Burden of valvular heart diseases: a population-based study. Lancet.

[89] Golbasi Z., Ucar O., Keles T. (2002). Increased levels of high sensitive C-reactive protein in patients with chronic rheumatic valve disease: evidence of ongoing inflammation. Eur J Heart Fail.

[90] Chandrashekhar Y., Westaby S., Narula J. (2009). Mitral stenosis. Lancet.

[91] Joswig B.C., Glover M.U., Handler J.B., Warren S.E., Vieweg W.V. (1982). Contrasting progression of mitral stenosis in Malayans versus American-born Caucasians. Am Heart J.

[92] Labovitz A.J., Nelson J.G., Windhorst D.M., Kennedy H.L., Williams G.A. (1985). Frequency of mitral valve dysfunction from mitral annular calcium as detected by Doppler echocardiography. Am J Cardiol.

[93] Ukita Y., Yuda S., Sugio H. (2016). Prevalence and clinical characteristics of degenerative mitral stenosis. J Cardiol.

[94] Serraf A., Zoghbi J., Belli E. (2000). Congenital mitral stenosis with or without associated defects: An evolving surgical strategy. Circulation.

[95] Ben Farhat M., Boussadia H., Gandjbakhch I. (1990). Closed versus open mitral commissurotomy in pure noncalcific mitral stenosis: hemodynamic studies before and after operation. J Thorac Cardiovasc Surg.

[96] Turi Z.G., Reyes V.P., Raju B.S. (1991). Percutaneous balloon versus surgical closed commissurotomy for mitral stenosis. A prospective, randomized trial. Circulation.

[97] Vahanian A. (2001). Balloon valvuloplasty. Heart.

[98] Fu J., Li Y., Zhang H. (2021). Outcomes of mitral valve repair compared with replacement for patients with rheumatic heart disease. J Thorac Cardiovasc Surg.

[99] Quentin V., Mesnier J., Delhomme C. (2023). Transcatheter mitral valve replacement using Transcatheter aortic valve or dedicated devices: current evidence and future prospects. J Clin Med.

[100] Scherman J., Ofoegbu C., Myburgh A. (2019). Preclinical evaluation of a transcatheter aortic valve replacement system for patients with rheumatic heart disease. EuroIntervention.

[101] Topilsky Y., Maltais S., Medina Inojosa J. (2019). Burden of tricuspid regurgitation in patients diagnosed in the community setting. JACC: Cardiovasc Imaging.

[102] Yang L., Chen H., Pan W. (2019). Analyses for prevalence and outcome of tricuspid regurgitation in China: an echocardiography study of 134,874 patients. Cardiology.

[103] Cahill T.J., Prothero A., Wilson J. (2021). Community prevalence, mechanisms and outcome of mitral or tricuspid regurgitation. Heart.

[104] Iung B., Delgado V., Rosenhek R. (2019). Contemporary presentation and management of valvular heart disease: the EURObservational Research Programme Valvular Heart Disease II Survey. Circulation.

[105] Hahn R.T., Lawlor M.K., Davidson C.J. (2023). Tricuspid valve academic research consortium definitions for tricuspid regurgitation and trial endpoints. Eur Heart J.

[106] Wang T.K.M., Akyuz K., Mentias A. (2022). Contemporary etiologies, outcomes, and novel risk score for isolated tricuspid regurgitation. JACC Cardiovasc Imaging.

[107] Patlolla S.H., Schaff H.V., Nishimura R.A. (2022). Incidence and burden of tricuspid regurgitation in patients with atrial fibrillation. J Am Coll Cardiol.

[108] Nishiura N., Kitai T., Okada T. (2023). Long-term clinical outcomes in patients with severe tricuspid regurgitation. J Am Heart Assoc.

[109] Topilsky Y., Nkomo V.T., Vatury O. (2014). Clinical outcome of isolated tricuspid regurgitation. JACC Cardiovasc Imaging.

[110] Kumar K., Byrne T., Simpson T.F. (2023). Clinical predictors of mortality and heart failure hospitalization in patients with severe tricuspid regurgitation. Structural Heart.

[111] Zack C.J., Fender E.A., Chandrashekar P. (2017). National trends and outcomes in isolated tricuspid valve surgery. J Am Coll Cardiol.

[112] Dreyfus J., Audureau E., Bohbot Y. (2022). TRI-SCORE: a new risk score for in-hospital mortality prediction after isolated tricuspid valve surgery. Eur Heart J.

[113] Kim D.Y., Kim J., Cho I. (2024). Validation of TRI-SCORE for outcome prediction after isolated tricuspid valve surgery in Asian patients. J Am Heart Assoc.

[114] Xu H., Chen M., Wang Z. (2024). Mid-term outcomes of K-Clip transcatheter tricuspid annuloplasty system in patients with severe functional tricuspid regurgitation. JACC Cardiovasc Interv.

[115] Zhang Y., Lu F., Li W. (2023). A first-in-human study of transjugular transcatheter tricuspid valve replacement with the LuX-Valve Plus system. EuroIntervention.

[116] Wang X., Ma Y., Liu Z. (2023). Comparison of outcomes between transcatheter tricuspid valve repair and surgical tricuspid valve replacement or repair in patients with tricuspid insufficiency. J Cardiothorac Surg.

[117] Wang Y., Liu Y., Meng X. (2024). Comparing outcomes of transcatheter tricuspid valve replacement and medical therapy for symptomatic severe tricuspid regurgitation: a retrospective study. Eur J Med Res.

[118] Dolk H., Loane M., Garne E., Group aESoCAW (2011). Congenital heart defects in Europe: prevalence and perinatal mortality, 2000 to 2005. Circulation.

[119] Van Der Linde D., Konings E.E., Slager M.A. (2011). Birth prevalence of congenital heart disease worldwide: a systematic review and meta-analysis. J Am Coll Cardiol.

[120] Liu Y., Chen S., Zühlke L. (2019). Global birth prevalence of congenital heart defects 1970–2017: updated systematic review and meta-analysis of 260 studies. Int J Epidemiol.

[121] Blue G.M., Kirk E.P., Giannoulatou E. (2017). Advances in the genetics of congenital heart disease: a clinician’s guide. J Am Coll Cardiol.

[122] Zhao L., Chen L., Yang T. (2020). Birth prevalence of congenital heart disease in China, 1980–2019: a systematic review and meta-analysis of 617 studies. Eur J Epidemiol.

[123] Ha K.S., Park C.M., Lee J. (2024). Nationwide birth prevalence of crucial congenital heart defects from 2014 to 2018 in Korea. Korean Circ J.

[124] Pan W., Zhou D., Hijazi Z.M. (2024). 2024 Statement from Asia expert operators on transcatheter pulmonary valve replacement. Catheter Cardiovasc Interv.

[125] Liu F., Yang Y.-N., Xie X. (2015). Prevalence of congenital heart disease in Xinjiang multi-ethnic region of China. PloS One.

[126] Li Y., Wei X., Zhao Z. (2017). Prevalence and complications of bicuspid aortic valve in Chinese according to echocardiographic database. Am J Cardiol.

[127] Sun B.J., Oh J.K., Lee S.H. (2019). Mid-term clinical outcomes in a cohort of asymptomatic or mildly symptomatic Korean patients with bicuspid aortic valve in a tertiary referral hospital. J Cardiovasc Imaging.

[128] Vervoort D., Meuris B., Meyns B., Verbrugghe P. (2020). Global cardiac surgery: access to cardiac surgical care around the world. J Thorac Cardiovasc Surg.

[129] Dib N., Chauvette V., Diop M.S. (2023). Tetralogy of Fallot in Low-and Middle-Income Countries. CJC Pediatr Congenit Heart Dis.

[130] Jin Q., Long Y., Zhang G. (2024). Five-year follow-up after percutaneous pulmonary valve implantation using the Venus P-valve system for patients with pulmonary regurgitation and an enlarged native right ventricular outflow tract. Catheter Cardiovasc Interv.

[131] Sengupta S.P., Prendergast B., Laroche C. (2023). Socioeconomic variations determine the clinical presentation, aetiology, and outcome of infective endocarditis: a prospective cohort study from the ESC-EORP EURO-ENDO (European Infective Endocarditis) registry. Eur Heart J Qual Care Clin Outcomes.

[132] Chen H., Zhan Y., Zhang K. (2022). The global, regional, and national burden and trends of infective endocarditis from 1990 to 2019: results from the global burden of disease study 2019. Front Med.

[133] Yang X., Chen H., Zhang D., Shen L., An G., Zhao S. (2022). Global magnitude and temporal trend of infective endocarditis, 1990–2019: results from the Global Burden of Disease Study. Eur J Prev Cardiol.

[134] Habib G., Erba P.A., Iung B. (2019). Clinical presentation, aetiology and outcome of infective endocarditis. Results of the ESC-EORP EURO-ENDO (European infective endocarditis) registry: a prospective cohort study. Eur Heart J.

[135] Li H.-L., Tromp J., Teramoto K. (2022). Temporal trends and patterns of infective endocarditis in a Chinese population: A territory-wide study in Hong Kong (2002–2019). Lancet Reg Health West Pac.

[136] Yamashita S., Tokushima M., Nakashima T., Katsuki N.E., Tago M., Yamashita S-i (2020). Clinical status quo of infective endocarditis in a university hospital in Japan: a single-hospital-based retrospective cohort study. Intern Med.

[137] Angsutararux T., Angkasekwinai N. (2019). Cumulative incidence and mortality of infective endocarditis in Siriraj hospital–Thailand: a 10-year retrospective study. BMC Infect Dis.

[138] Kim J.H., Lee H.J., Ku N.S. (2021). Infective endocarditis at a tertiary care hospital in South Korea. Heart.

[139] Abdulhak A.A.B., Baddour L.M., Erwin P.J. (2014). Global and regional burden of infective endocarditis, 1990–2010: a systematic review of the literature. Global Heart.

[140] Sunil M., Hieu H.Q., Arjan Singh R.S., Ponnampalavanar S., Siew K.S., Loch A. (2019). Evolving trends in infective endocarditis in a developing country: a consequence of medical progress?. Ann Clin Microbiol Antimicrob.

[141] Nakatani S., Mitsutake K., Ohara T. (2013). Recent picture of infective endocarditis in Japan–lessons from cardiac disease registration (CADRE-IE). Circ J.

[142] Hase R., Otsuka Y., Yoshida K., Hosokawa N. (2015). Profile of infective endocarditis at a tertiary-care hospital in Japan over a 14-year period: characteristics, outcome and predictors for in-hospital mortality. Int J Infect Dis.

[143] Yew H.S., Murdoch D.R. (2012). Global trends in infective endocarditis epidemiology. Curr Infect Dis Rep.

[144] Yiu K.-H., Siu C.-W., Lee K.L.-F. (2007). Emerging trends of community acquired infective endocarditis. Int J Cardiol.

[145] Watt G., Pachirat O., Baggett H.C. (2014). Infective endocarditis in northeastern Thailand. Emerg Infect Dis.

[146] IBRAHIM K.S., ISMAIL J.R., YUSOF Y. (2017). Pattern and predictors of outcomes for infective endocarditis in North Kuala Lumpur. Heart India.

[147] Wu Z., Chen Y., Xiao T., Niu T., Shi Q., Xiao Y. (2020). Epidemiology and risk factors of infective endocarditis in a tertiary hospital in China from 2007 to 2016. BMC Infect Dis.

[148] Xu H., Cai S., Dai H. (2016). Characteristics of infective endocarditis in a tertiary hospital in East China. PloS One.

[149] Ngiam J.N., Koh M.C.Y., Archuleta S. (2024). Performance of risk scores in predicting infective endocarditis in patients with Staphylococcus aureus bacteraemia in a prospective Asian cohort. J Clin Med.

[150] Tubiana S., Duval X., Alla F. (2016). The VIRSTA score, a prediction score to estimate risk of infective endocarditis and determine priority for echocardiography in patients with Staphylococcus aureus bacteremia. J Infect.

[151] Kui S., Huang W., Tan W., Chai S. (2020). P266 Infective endocarditis: exploring geographical trends and comparing risk models in a multi-ethnic Asian population. Eur Heart J.

[152] Chobufo M.D., Atti V., Vasudevan A. (2023). Trends in infective endocarditis mortality in the United States: 1999 to 2020: a cause for alarm. J Am Heart Assoc.

[153] Talha K.M., Baddour L.M., Thornhill M.H. (2021). Escalating incidence of infective endocarditis in Europe in the 21st century. Open Heart.

[154] Talha K.M., Dayer M.J., Thornhill M.H. (2021). Temporal trends of infective endocarditis in North America from 2000 to 2017—a systematic review. Open Forum Infect Dis.

[155] Slipczuk L., Codolosa J.N., Davila C.D. (2013). Infective endocarditis epidemiology over five decades: a systematic review. PloS One.

[156] Poesen K., Pottel H., Colaert J., Niel C.D. (2014). Epidemiology of infective endocarditis in a large Belgian non-referral hospital. Acta Clinica Belgica.

[157] Becher P.M., Gossling A., Fluschnik N. (2024). Temporal trends in incidence, patient characteristics, microbiology and in-hospital mortality in patients with infective endocarditis: a contemporary analysis of 86,469 cases between 2007 and 2019. Clin Res Cardiol.

[158] Iung B., Baron G., Butchart E.G. (2003). A prospective survey of patients with valvular heart disease in Europe: The Euro Heart Survey on Valvular Heart Disease. Eur Heart J.

[159] Duan Z., Ye Y., Li Z. (2024). Contemporary spectrum, characteristics, and outcomes of adult patients with rheumatic valvular disease in China: insights from the China-VHD study. Int J Cardiol. Cardiovasc Risk Prev.

[160] Unger P., Clavel M.A., Lindman B.R., Mathieu P., Pibarot P. (2016). Pathophysiology and management of multivalvular disease. Nat Rev Cardiol.

[161] Writing Committee M., Otto C.M., Nishimura R.A. (2021). 2020 ACC/AHA guideline for the management of patients with valvular heart disease: a report of the American College of Cardiology/American Heart Association Joint Committee on Clinical Practice Guidelines. J Am Coll Cardiol.

[162] Lamichhane P., Pokhrel K.M., Pokharel P., Bhandari B., Lamichhane P., Regmi P.R. (2022). Prevalence of rheumatic heart disease in South Asia: a systematic review and meta-analysis. Int J Cardiol.

[163] Godown J., Lu J.C., Beaton A. (2015). Handheld echocardiography versus auscultation for detection of rheumatic heart disease. Pediatrics.

[164] Cohen-Shelly M., Attia Z.I., Friedman P.A. (2021). Electrocardiogram screening for aortic valve stenosis using artificial intelligence. Eur Heart J.

[165] Faletra F.F., Agricola E., Flachskampf F.A. (2023). Three-dimensional transoesophageal echocardiography: how to use and when to use-a clinical consensus statement from the European Association of Cardiovascular Imaging of the European Society of Cardiology. Eur Heart J Cardiovasc Imaging.

[166] Yap J., Hayashida K., Lee M.K.Y. (2024). Asian Pacific Society of Cardiology position statement on the use of transcatheter aortic valve implantation in the management of aortic stenosis. JACC Asia.

[167] Lancellotti P., Pellikka P.A., Budts W. (2017). The clinical use of stress echocardiography in non-ischaemic heart disease: recommendations from the European Association of Cardiovascular Imaging and the American Society of Echocardiography. J Am Soc Echocardiogr.

[168] Raja Shariff R.E., Soesanto A.M., Scalia G.M. (2023). Echocardiographic imaging in transcatheter structural intervention: an AAE review paper. JACC Asia.

[169] Blanke P., Weir-McCall J.R., Achenbach S. (2019). Computed tomography imaging in the context of transcatheter aortic valve implantation (TAVI)/transcatheter aortic valve replacement (TAVR): an expert consensus document of the Society of Cardiovascular Computed Tomography. JACC Cardiovasc Imaging.

[170] Giannini F., Baldetti L., Gallone G., Tzanis G., Latib A., Colombo A. (2018). Transcatheter valve replacement in Asia Pacific: current practice and perspectives. J Am Coll Cardiol.

[171] Jilaihawi H., Wu Y., Yang Y. (2015). Morphological characteristics of severe aortic stenosis in China: imaging corelab observations from the first Chinese transcatheter aortic valve trial. Catheter Cardiovasc Interv.

[172] So KC-y, Xu J., Kam KK-h (2025). Current status of tricuspid valve interventions in Asia Pacific region. JACC Asia.

[173] Tang G.H.L., Zaid S., Hahn R.T. (2025). Structural heart imaging using 3-dimensional intracardiac echocardiography: JACC Cardiovascular Imaging Position Statement. JACC Cardiovasc Imaging.

[174] Jingquan Z., Deyong L., Huimin C. (2022). Intracardiac echocardiography Chinese expert consensus. Front Cardiovasc Med.

[175] Vahanian A., Beyersdorf F., Praz F. (2021). 2021 ESC/EACTS guidelines for the management of valvular heart disease: developed by the Task Force for the Management of Valvular Heart Disease of the European Society of Cardiology (ESC) and the European Association for Cardio-Thoracic Surgery (EACTS). Eur Heart J.

[176] Tam D.Y., Rocha R.V., Wijeysundera H.C., Austin P.C., Dvir D., Fremes S.E. (2020). Surgical valve selection in the era of transcatheter aortic valve replacement in the Society of Thoracic Surgeons Database. J Thorac Cardiovasc Surg.

[177] Kim H.J., Kang D.Y., Park H. (2021). Comparison of sutureless bioprosthetic valve with surgical or TAVR for severe aortic stenosis. JACC Asia.

[178] Al-Abcha A., Saleh Y., Boumegouas M. (2021). Meta-analysis of valve-in-valve transcatheter aortic valve implantation versus redo-surgical aortic valve replacement in failed bioprosthetic aortic valve. Am J Cardiol.

[179] Bermejo J., Postigo A., Baumgartner H. (2021). The year in cardiovascular medicine 2020: valvular heart disease. Eur Heart J.

[180] Giannini F., Baldetti L., Gallone G., Tzanis G., Latib A., Colombo A. (2018). Transcatheter Valve replacement in Asia Pacific: current practice and perspectives. J Am Coll Cardiol.

[181] Miyasaka M., Tada N., Taguri M. (2018). Incidence, predictors, and clinical impact of prosthesis-patient mismatch following transcatheter aortic valve replacement in Asian patients: the OCEAN-TAVI registry. JACC Cardiovasc Interv.

[182] Yoon S.-H., Ahn J.-M., Hayashida K. (2016). Clinical outcomes following transcatheter aortic valve replacement in Asian population. JACC Cardiovasc Interv.

[183] Tay E., Khaing T., Yin W.H. (2021). Asia Pacific TAVI registry (an APSIC initiative): initial report of early outcomes: Asia Pacific TAVI registry. AsiaIntervention.

[184] Yamamoto Y., Iino K., Shintani Y. (2017). Comparison of aortic annulus dimension after aortic valve neocuspidization with valve replacement and normal valve. Semin Thorac Cardiovasc Surg.

[185] Franzen O., Baldus S., Rudolph V. (2010). Acute outcomes of MitraClip therapy for mitral regurgitation in high-surgical-risk patients: emphasis on adverse valve morphology and severe left ventricular dysfunction. Eur Heart J.

[186] Tamburino C., Ussia G.P., Maisano F. (2010). Percutaneous mitral valve repair with the MitraClip system: acute results from a real world setting. Eur Heart J.

[187] Franzen O., van der Heyden J., Baldus S. (2011). MitraClip® therapy in patients with end-stage systolic heart failure. Eur J Heart Fail.

[188] Schillinger W., Senges J. (2013). [TRAMI (Transcatheter Mitral Valve Interventions) register. The German mitral register]. Herz.

[189] Sürder D., Klersy C., Corti R. (2020). Impact of mitral regurgitation aetiology on MitraClip outcomes: the MitraSwiss registry. EuroIntervention.

[190] Yeo K.K., Wong N. (2020). Percutaneous edge-to-edge mitral valve repair. Korean Circ J.

[191] Feldman T., Foster E., Glower D.D. (2011). Percutaneous repair or surgery for mitral regurgitation. N Engl J Med.

[192] Hayashida K., Yasuda S., Matsumoto T. (2017). AVJ-514 trial-baseline characteristics and 30-day outcomes following MitraClip(®) treatment in a Japanese Cohort. Circ J.

[193] Lee C.W., Sung S.H., Tsai Y.L., Chang T.Y., Hsu C.P., Lu C.C. (2018). Initial experience with percutaneous edge-to-edge transcatheter mitral valve repair in a tertiary medical center in Taiwan. J Chin Med Assoc.

[194] Structural Heart Disease Professional Committee Cardiovascular Branch CPA (2020). Chinese expert consensus on transcatheter aortic valve replacement (2020 update). Chin J Intervent Cardiol.

[195] Gupta P., Arora S., Qamar A., Gupta M., Seth A. (2019). Current status of transcatheter aortic valve replacement in India. Cardiovasc Diagn Ther.

[196] Garcia S., Elmariah S., Cubeddu R.J. (2024). Mitral transcatheter edge-to-edge repair with the PASCAL precision system: device knobology and review of advanced steering maneuvers. Structural Heart.

[197] Lim D.S., Kar S., Spargias K. (2019). Transcatheter valve repair for patients with mitral regurgitation: 30-day results of the CLASP study. JACC Cardiovasc Interv.

[198] Jin Q.W., Mohd Ghazi A.B., Kolanthaivelu J., Azmi Yahaya S. (2022). Novel treatment of atrial functional tricuspid regurgitation using transcatheter bicaval valve implantation (TricValve). AsiaIntervention.

[199] Mahboob E., Samad M.A., Carver C. (2024). TriClip G4: A game-changer for tricuspid valve regurgitation treatment. Curr Prob Cardiol.

[200] Sorajja P., Whisenant B., Hamid N. (2023). Transcatheter repair for patients with tricuspid regurgitation. N Engl J Med.

[201] Chiam P.T. (2018). Transcatheter aortic valve implantation in Asia: the first decade. EuroIntervention.

[202] Khan M.S., Shahid I., Bennis A., Rakisheva A., Metra M., Butler J. (2024). Global epidemiology of heart failure. Nat Rev Cardiol.

[203] Li H., Song X., Liang Y. (2022). Global, regional, and national burden of disease study of atrial fibrillation/flutter, 1990-2019: results from a global burden of disease study, 2019. BMC Pub Health.

[204] Mond H.G., Crozier I., Sloman J.G. (2023). The Australian and New Zealand cardiac implantable electronic device survey, calendar year 2021: 50-year anniversary. Heart Lung Circ.

[205] Zoghbi W.A., Levine R.A., Flachskampf F. (2022). Atrial functional mitral regurgitation: a JACC: cardiovascular imaging expert panel viewpoint. JACC Cardiovasc Imaging.

[206] Yu C.W., Kim W.J., Ahn J.M. (2018). Trends and outcomes of transcatheter aortic valve implantation (TAVI) in Korea: the results of the first cohort of Korean TAVI Registry. Korean Circ J.

[207] Maznyczka A., Heg D., Tomii D. (2024). Asymmetrical expansion of balloon-expandable transcatheter aortic valve prostheses: implications for valve hemodynamic and clinical outcomes. JACC Cardiovasc Interv.

[208] Tang G.H.L., Hooda A., Zaid S. (2022). Outcomes and feasibility of redo-TAVR after Sapien 3 Ultra TAVR in extremely-undersized versus nominally-sized annuli. Catheter Cardiovasc Interv.

